# Morphology of the Bony Labyrinth Supports the Affinities of *Paradolichopithecus* with the Papionina

**DOI:** 10.1007/s10764-022-00329-4

**Published:** 2022-09-20

**Authors:** Anne Le Maître, Franck Guy, Gildas Merceron, Dimitris S. Kostopoulos

**Affiliations:** 1 Department of Evolutionary Biology, University of Vienna, Djerassiplatz 1, 1030 Vienna, Austria; 2 PALEVOPRIM – UMR 7262 CNRS INEE, Université de Poitiers, Poitiers, France; 3 Laboratory of Geology and Palaeontology, Aristotle University of Thessaloniki, Thessaloniki, Greece

**Keywords:** *Paradolichopithecus*, Papionini, Early Pleistocene, Dafnero, Greece, Phylogeny, Bony labyrinth

## Abstract

Discoveries in recent decades indicate that the large papionin monkeys *Paradolipopithecus* and *Procynocephalus* are key members of the Late Pliocene – Early Pleistocene mammalian faunas of Eurasia. However, their taxonomical status, phylogenetic relationships, and ecological profile remain unclear. Here we investigate the two latter aspects through the study of the inner ear anatomy, as revealed by applying micro-CT scan imaging techniques on the cranium LGPUT DFN3-150 of *Paradolichopithecus* from the lower Pleistocene (2.3 Ma) fossil site Dafnero-3 in Northwestern Greece. Using geometric morphometric methods, we quantified shape variation and the allometric and phylogenetic signals in extant cercopithecines (*n* = 80), and explored the morphological affinities of the fossil specimen with extant taxa. LGPUT DFN3-150 has a large centroid size similar to that of baboons and their relatives. It shares several shape features with Macacina and Cercopithecini, which we interpret as probable retention of a primitive morphology. Overall, its inner ear morphology is more consistent with a stem Papionini more closely related to Papionina than Macacina, or to a basal crown Papionina. Our results, along with morphometrical and ecological features from previous studies, call into question the traditional hypothesis of a *Paradolichopithecus*-Macacina clade, and provide alternative perspectives in the study of Eurasian primate evolution during the late Neogene-Quaternary.

**Supplementary Information:**

The online version contains supplementary material available at 10.1007/s10764-022-00329-4.

## Introduction

At the dawn of the Quaternary and as the Pliocene forests shrank in favor of Pleistocene grasslands under successively cooler climatic conditions (e.g., Popescu *et al*., 2010), a remarkably homogeneous mammal fauna appears across Eurasia. Stenonoid horses, leptobovines, gazelles, and a variety of cervids are associated with machairodontines, running hyaenas, and raccoon-dogs, the latter species soon replaced by dog-like carnivores (e.g., Aguirre *et al*., 1994; Agusti & Antón, 2002; Hermier *et al*., 2020; Koufos, 2001; Koufos *et al*., 2020; Qiu *et al*., 2004; Sotnikova & Rook, 2010). Within this transitional context, primates are represented by the supposedly forest-dependent macaques (genus *Macaca* Lacépède, 1799) and the presumed open-dweller large monkeys referred to the genera *Procynocephalus* Schlosser, 1924 and *Paradolichopithecus* Necrasov, Samson, & Radulesco, 1961.

The Late Pliocene to Early Pleistocene taxon *Procynocephalus* was the first fossil primate described; but it is still poorly known, mainly from China and India (Baker & Durand, 1836; Szalay & Delson, 1979; Takai *et al*., 2014). *Paradolichopithecus*, in contrast, is recorded in France, Spain, Romania, Serbia, Greece, Tajikistan, and China (Eronen & Rook, 2004; Kostopoulos *et al*., 2018; Radović *et al*., 2019; Szalay & Delson, 1979 and ref. therein) and its emergence appears to predate that of *Procynocephalus*, with the earliest occurrences being dated around 3.2 Ma. Both genera experienced the maximum of their geographic distribution during the same time interval (ca. 3.0–2.0 Ma), and across the same Eurasian latitudinal belt (roughly within 35°–45°; Takai *et al*., 2008: Fig. 7). Some authors hypothesize they are synonymous (e.g., Kostopoulos *et al*., 2018; Nishimura *et al*., 2010 and ref. therein); others stress, however, the paucity of the available material for formal decisions (e.g., Jablonski, 2002; Szalay & Delson, 1979). Both genera are traditionally considered as representing large macacinans (Delson & Frost, 2004; Strasser & Delson, 1987), although other authors propose closer phylogenetic affinities with the African baboons (Jolly, 1967; Kostopoulos *et al*., 2018; Maschenko, 1994; Takai *et al*., 2008).

The estimated body mass of *Paradolichopithecus* (ca. 17 kg for females and 40 kg for males; Kostopoulos *et al*., 2018: Fig. 10) significantly exceeds known ranges of fossil and extant *Macaca*, indicating this taxon entered into novel size classes, comparable to those of extant baboons and relatives. An evolutionary increase in body size is reported to be the main force toward new cranial shape patterns gained allometrically in Papionina (Frost *et al*., 2003; Gilbert & Rossie, 2007; Gilbert *et al*., 2009; Joganic & Heuzé, 2019; Joganic *et al*., 2018; Leigh, 2006; Leigh *et al*., 2003; Monson *et al*., 2017; Nishimura *et al*., 2019; Profico *et al*., 2017; Singleton, 2002). Large size in *Paradolichopithecus* is associated with specific postcranial adaptations (e.g., Sondaar *et al*., 2006; Szalay & Delson, 1979; Ting *et al*., 2004; van der Geer & Sondaar, 2002) highlighting a terrestrial way of life comparable to that of baboons, and in accordance with available information from dental tissue (Plastiras, 2021; Williams & Holmes, 2012). Whether, however, this common path to increased size and terrestrial behaviors reflects close phylogenetic relationships between *Paradolichopithecus* and baboons or it simply represents an example of parallelism still has to be tested.

**Fig. 1:**
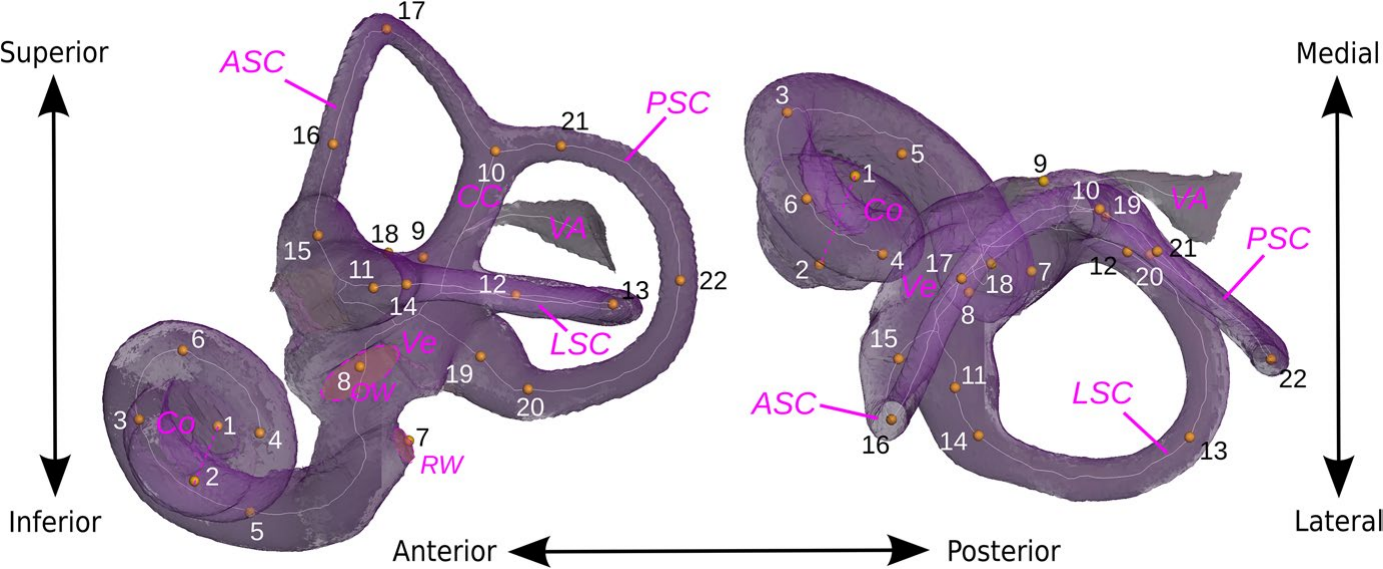
Position of 22 landmarks on the left bony labyrinth of Macaca fascicularis MCZ-23812 in lateral (*left*) and superior (*right*) views. The landmarks are defined following Lebrun *et al*. (2010), as detailed in Appendix S2. *ASC*, anterior semicircular canal; *CC*, common crus; *Co*, cochlea; *LSC*, lateral semicircular canal; *OW*, oval window; *PSC*, posterior semicircular canal; *RW*, round window; *VA*, vestibular aqueduct; *Ve*, vestibule. The *dashed line* corresponds to the axis of the cochlea

Given the persistent controversy about the phylogenetic position of *Paradolichopithecus*, the importance of the evidence coming from phylogenetically informative anatomical areas is paramount. In vertebrates, the inner ear is involved in many functions: hearing, balance, posture control, and gaze stabilization (e.g., Graf & Klam, 2006). The cochlear system enables sound perception, while the vestibular system detects head movements and accelerations (Fig. 1). The vestibular system corresponds to the semicircular canal system and the vestibule. The semicircular canal system in composed of the lateral, anterior, and posterior semicircular canals (respectively LSC, ASC and PSC), the two latter being connected at the common crus. The circular movements of the head are detected at the bulging base of each canal (the ampulla). The vestibule (Ve) houses the utricle and the saccule, the two otolith organs of the ear that detect linear movements and accelerations, including gravity. Sounds are detected in the cochlea (Co), coiled around an axis. The sound waves are propagated through the oval window (OW); they propagate along the coils of the cochlea until its apex, then back to its base until they reach the round window (RW), where they dissipate. The vestibular aqueduct (VA) is connected to the vestibule, and is involved in hearing. Since it is made of soft tissue, the inner ear is not preserved in fossil specimens. However it is possible to study the bony labyrinth, its osseous surroundings in the thick petrous part of the temporal bone, because its shape roughly mimics the inner ear. Bony labyrinth anatomy has previously been demonstrated to provide phylogenetic, functional, and paleoecological signals.

In mammals, the semicircular canal morphology was found to be linked to agility (Spoor *et al*., 2007; Silcox *et al*., 2009; Perier *et al*., 2016; Gonzales *et al*., 2019; *contra* Morimoto *et al*., 2020), posture (Le Maître *et al*., 2017; Spoor & Zonneveld, 1998; Spoor *et al*., 1994, 1996), locomotion (Georgi, 2008; but see Rae *et al*., 2016), and ecology (Grohé *et al*., 2016; Pfaff *et al*., 2015; Ekdale, 2016; Schwab *et al*., 2019). Recent studies have highlighted the importance of taking intraspecific variation into account in analyses of the adaptive signal, and not just the average morphology (Gonzales *et al*., 2019; Lebrun *et al*., 2021).

In primates, the morphology of the labyrinth is phylogenetically informative at high taxonomic levels (orders, infraorders, superfamilies, or families, see Lebrun *et al*., 2010, 2012; Morimoto *et al*., 2020). At lower taxonomic levels, genera, species, and subspecies can be discriminated in anthropoid primates based on labyrinth morphology, even if the exact pattern of phylogenetic relationships is not completely reflected (Spoor, 1993; Gunz *et al*., 2012; Beaudet *et al*., 2016; Urciuoli *et al*., 2020, Urciuoli & Zanolli, 2021; del Rio *et al*., 2021). Therefore, the bony labyrinth can be used to reconstruct the locomotor behavior of a fossil species or to assess its phylogenetic relationships with other taxa - at least at suprageneric taxonomic ranks, although discriminating between these two lines of evidence is not always straightforward.

The morphology of the labyrinth also reflects body dimensions. Heavier primates tend to have a larger bony labyrinth in absolute size, but smaller relative to their body mass (e.g., Spoor & Zonneveld, 1998; Spoor *et al*., 2007; Walker *et al*., 2008; Silcox *et al*., 2009; Ryan *et al*., 2012). A small but statistically significant fraction of the shape of the bony labyrinth is predicted by body mass (del Rio *et al*., 2021) or by labyrinth size (Le Maître *et al*., 2017; Urciuoli *et al*., 2020). Available evidence shows that in mammals, labyrinth size and shape change barely after petrous bone ossification, which happens before birth, at least in humans (Jeffery & Spoor, 2004; Mejdoubi *et al*., 2015, 2016; *contra* Boucherie *et al*., 2021) and ruminants (Maier, 2013; Mennecart & Costeur, 2016). Therefore, labyrinth morphological variation is likely to preclude size and shape variation involving biological mechanisms for which the onset occurs after the definitive size is acquired (e.g., allometric shape changes of the skull, sexual dimorphism). Because phylogeny, body mass, and ecology are not totally independent in primates, disentangling these three elements in labyrinth morphology is even more difficult.

In the present study, we attempt to clarify the phylogenetic affinities of the fossil genus *Paradolochopithecus*, based on the bony labyrinth of the cranium LGPUT DFN3-150 of *Paradolichopithecus* aff. *arvernensis* from the Lower Pleistocene site of Dafnero-3 in Northwestern Greece, identified as a subadult female (Kostopoulos *et al*., 2018). For this purpose, we analyse the morphology of this structure, reconstructed from micro-CT scan imaging, using geometric morphometric methods. We investigate the potential of bony labyrinth morphology to reconstruct phylogenetic relationships within Cercopithecinae, and we assess the impact of allometry on labyrinth shape. Based on these elements, we use the bony labyrinth to clarify the affinities of the fossil with Macacina and Papionina, the two extant subtribes of the cercopithecine tribe Papionini.

As body mass is highly variable across Cercopithecinae, we anticipate that size is the main component of morphological variation for the bony labyrinth (hypothesis H1), so we predict a larger labyrinth for larger primates (prediction P1). Because allometry is known to be an important component of skull shape variation in this group (Frost *et al*., 2003; Joganic & Heuzé, 2019; Joganic *et al*., 2018; Nishimura *et al*., 2019; Profico *et al*., 2017), we propose the hypothesis that this also applies to the inner ear (H2). Therefore, we predict that the main component of shape variation is allometry (P2). If the first hypothesis (H1) is supported, then we predict that because of its heavy body mass (ca. 19.5 kg; Kostopoulos *et al*., 2018), the bony labyrinth of the fossil specimen of *Paradolichopithecus* would be large and, together with H2, it would imply a shape similar to that of large Papionina. Based on previous studies in related primates, we propose the hypothesis that the labyrinth morphology of Cercopithecinae reflects both phylogeny and ecology (H3). We do not specifically test the adaptive signal in the present study, but we can predict from H3 that we will find a phylogenetic signal in labyrinth morphology (P3a), and that because it is blurred by the adaptive signal, labyrinth morphology better reflects phylogeny at high taxonomic levels (P3b). However, as body mass in Papionina tends to be larger than in Macacina and Cercopithecini, we suppose that the phylogenetic signal in the bony labyrinth morphology is mainly driven by size (H4), which predicts a weaker signal when size effects are removed (P4). If the two hypotheses regarding the phylogenetic signal (H3 and H4) are true, then we can use the labyrinth morphology as an indicator of the phylogenetic affinities of the fossil. However, if H2 is true, we should preferentially remove the allometric component of shape, to avoid size effects.

## Methods

### Sample Composition

The fossil cranium LGPUT DFN3-150 of *Paradolichopithecus aff. arvernensis* is housed in the Museum of Geology–Palaeontology–Palaeoanthropology of the Aristotle University of Thessaloniki (LGPUT). We collected high-resolution micro-computed tomography (HR-μCT) images at the PLATeforme INstrumentale d’Analyses – PLATINA (IC2MP, Université de Poitiers). The protocol followed and the technical parameters are given in Appendix S1 and by Kostopoulos *et al*. (2018).

The comparative sample for the morphometric study of the labyrinth (Table I; Appendix S1: table) consisted of μCT-scans of the dry skulls of 80 specimens representing 25 extant species in ten cercopithecine genera. Because *Paradolichopithecus* is thought to be a Papionini, we mainly included specimens from this tribe: 35 Papionina (six specimens of *Cercocebus*, six *Lophocebus*, eight *Mandrillus*, ten *Papio*, five *Theropithecus*) and 32 Macacina (genus *Macaca*). For comparisons, we added 13 specimens representing four genera of Cercopithecini (one *Allochrocebus*, two *Cercopithecus*, seven *Chlorocebus*, and three *Erythrocebus*). Thirty-four specimens are females, 39 specimens are males, while the sex was not available for the rest of the sample. Twenty-five individuals are juveniles or young adults (as their third molars are not fully erupted), but they can be included in the comparison, since maturity does not affect the labyrinthine morphology (Jeffery & Spoor, 2004).

**Table I:** Summary of the sample used for the study of the bony labyrinth of the fossil specimen, compared to extant species (*n* = 80). Papionina and Macacina are two subtribes of tribe Papionini, the sister group of the tribe Cercopithecini. See appendix S1 for more details

**Species**	**Sex**^a^			**Total**
	F	M	NA	
**Fossil** (*n* = 1)				
*Paradolichopithecus* aff. *arvernensis*	1	0	0	**1**
**Cercopithecini** (*n* = 13)				
*Allochrocebus lhoesti*	0	1	0	**1**
*Cercopithecus cephus*	1	1	0	**2**
*Chlorocebus aethiops*	1	2	1	**4**
*Chlorocebus pygerythrus*	2	1	0	**3**
*Erythrocebus patas*	1	2	0	**3**
**Papionina** (*n* = 35)				
*Cercocebus agilis*	2	1	0	**3**
*Cercocebus atys*	0	1	0	**1**
*Cercocebus torquatus*	1	1	0	**2**
*Lophocebus albigena*	4	2	0	**6**
*Mandrillus leucophaeus*	1	3	2	**6**
*Mandrillus sphinx*	0	2	0	**2**
*Papio anubis*	5	1	0	**7**
*Papio cynocephalus*	1	1	0	**2**
*Papio hamadryas*	0	1	0	**1**
*Theropithecus gelada*	2	3	0	**5**
**Macacina** (*n* = 32)				
*Macaca fascicularis*	6	3	2	**11**
*Macaca fuscata*	2	0	1	**3**
*Macaca hecki*	2	0	0	**2**
*Macaca leonina*	1	0	0	**1**
*Macaca maura*	0	1	0	**1**
*Macaca mulatta*	2	2	0	**4**
*Macaca nigra*	1	0	0	**1**
*Macaca radiata*	1	0	0	**1**
*Macaca* sp.	0	1	0	**1**
*Macaca sylvanus*	1	4	0	**5**
*Macaca thibetana*	1	0	1	**2**

^a^ Sex: F, female; M, male; NA, not available

### Data Acquisition

We downloaded the microCT-scans of 37 primate skulls from the MorphoSource website (www.MorphoSource.org, Duke University), which have resolutions ranging from 58.7 to 123.3 μm (isometric voxel size). We also scanned 15 skulls using an X-ray microtomograph at the IC2MP, Université de Poitiers, at resolutions ranging from 13.0 to 74.0 μm (isometric voxel size): six specimens are curated by the Royal Museum of Central Africa, Tervuren, Belgium, one by PALEVOPRIM lab, and eight by the Muséum National d’Histoire Naturelle, Paris, France. Finally, we directly used the 3D surface of the bony labyrinth for 28 specimens from three different museums, which were scanned at resolutions ranging from 33 to 96 μm (isometric voxel size), and virtually extracted by Beaudet *et al*., (2016, 2019; personal communication; see Appendix S1 for more details).

We virtually extracted the bony labyrinth on one side (preferentially left) from the microCT-scans using Amira (Thermo Fisher Scientific) software, and processed the generated surfaces using GeomagicStudio (Geomagic, Inc.) software to remove artifacts (little holes and spikes, overlapping mesh triangles). After calculating the centerline of the 3D volume (using the ‘auto skeleton’ tool in Amira), we positioned 22 landmarks on the surface and the centerline of the labyrinth (Lebrun *et al*., 2010; Le Maître, 2019; Fig. 1; see Appendix S2 for landmark definitions, and Appendix S3 for raw landmark coordinates). When we extracted the right labyrinth, we mirrored the surface using GeomagicStudio software for comparisons with the left side. For the fossil specimen, we extracted both left and right sides (Appendix S4-1 and S4-2).

### Analyses

#### Allometry

Using R v4.1.1 software (R Development Core Team, 2021), we aligned, rotated, and scaled the 3D landmark coordinates by a Procrustes superimposition (Bookstein, 1991; Rohlf & Slice, 1990). To assess the effect of body mass on labyrinth size, we did an ordinary least squares (OLS) regression of labyrinth centroid size on body mass, in log scale. We also tested the effect of body mass on labyrinth shape using a multivariate regression of the Procrustes shape coordinates on the natural logarithm of body mass. Because we found an association between body mass and labyrinth centroid size, we also tested the joint effect of body mass and centroid size on labyrinth shape, using a regression of the Procrustes shape coordinates on both variables expressed in log scale, with and without interaction effects. We conducted all analyses on the 79 extant specimens (without the fossil and the undefined *Macaca*). As body mass was not available for each individual, we used for each species the arithmetic mean body mass for a male or a female (Delson *et al*., 2000). When the sex was not determined, we excluded the observation.

To quantify the allometric effects within the bony labyrinth, we performed a multivariate regression of the Procrustes shape coordinates on the natural logarithm of centroid size (Bookstein, 1991; Mitteroecker *et al*., 2013), using an ordinary least squares estimation method. We performed a permutation test with 10,000 iterations against the null hypothesis of independence between the size and shape variables. We visualised the regression score as a function of log centroid size (CS). The regression score corresponds to the shape variable that is most strongly associated with the independent variable, here log CS ("shape score [defined] by projecting the shape data onto a line in the direction of the regression vector for the independent variable"; Drake & Klingenberg, 2008). As it has been argued that *Paradolichopithecus* might be a large macaque (Delson & Frost, 2004; Strasser & Delson, 1987), we visualised the line corresponding to the ordinary least squares regression of the regression score on log centroid size for the whole sample, and for three clades: Cercopithecini, Papionina, and Macacina (the two latter representing together the extant Papionini subtribes). We used the same clades to compute the bgPCA (see below). From this, we could see the regression score predicted for a primate having the same CS as the fossil LGPUT DFN3-150, to check whether its morphological similarities with baboons might be due to allometry. The prediction is much less reliable for the regression based on Macacina compared to Papionina (and Cercopithecini), because the former is an extrapolation (estimation beyond the observation range for Macacina), whereas the latter is an interpolation (estimation within the observation range for Papionina and Cercopithecini).

#### Morphological Variation

To explore the morphological diversity of the inner ear across cercopithecines, we performed a principal component analysis (PCA) on all specimens. We used the Procrustes shape coordinates as variables. To check whether any principal component corresponds to the allometric signal, we assessed the strength of the association between each PC and centroid size. For comparison, we also conducted a PCA on the residuals of the regression of Procrustes shape coordinates on log centroid size. As the results were very similar for both principal component analyses, we only present the results for Procrustes shape coordinates in the main text, and we used these variables for all further analyses. We performed all analyses using the *MASS* (Venables & Ripley, 2002), *geomorph* 4.0.3 (Adams *et al*., 2021; Baken *et al*., 2021) and *Morpho* 2.9 (Schlager, 2017) packages in R, and visualised the 3D shape changes associated with the principal components as warped surfaces using the *rgl* 0.106.8 (Murdoch & Adler, 2021) and *Morpho* packages.

To get a better separation among the three clades (Cercopithecini, Papionina, and Macacina), we performed a between-group principal component analysis (bgPCA). We preferred this type of analysis, rather than a canonical variate analysis, because the conditions of multivariate normality and homoscedasticity are unlikely to be met (Boulesteix, 2005; Mitteroecker & Bookstein, 2011). As the number of morphological variables is high relative to the number of cases per group, we conducted the analyses on reduced data to avoid spurious group discrimination (see Bookstein, 2019; Cardini & Polly, 2020 for caution on bgPCA): the first seven principal components (53.4% of the explained variance) obtained from the PCA based on the Procrustes shape coordinates. The number of principal components corresponds to the minimum number of PCs required to get the best classification rate. We performed a leave-one-out cross-validation to test the robustness of the prediction. We projected the position of the fossil in the morphological space of the between-group principal components (bgPCs). We also computed the typicality probabilities for the fossil to belong to each clade, based on the bgPCs. For the computation of typicality probabilities, we adjusted Mahalanobis D^2^ for small sample sizes as suggested by Wilson (1981). We performed the analyses using the functions “groupPCA”, “predict.bgPCA”, and "typprobClass" of the package *Morpho* (Schlager, 2017).

#### Phylogenetic Signal

Using the “nj” function of the package *ape* 5.0 (Paradis & Schliep, 2019), we performed neighbor-joining clustering analyses based on the Euclidean distances among Cercopithecinae species (Saitou & Nei, 1987). We computed the Euclidean distances based on form (Procrustes shape coordinates and log CS), shape (Procrustes shape coordinates), and allometry-free shape (regression residuals). The Euclidean distances between Procrustes shape coordinates correspond to Procrustes distances.

We tested the phylogenetic signal using the *Κ*-statistic (Blomberg *et al*., 2003) and its multivariate version the *Κ*_mult_ statistic (Adams, 2014), on the mean labyrinth morphology of each extant species, for the whole sample and for Papionini only (we excluded the *Macaca* specimen without species identification). We tested the signal for centroid size (= size), for regression residuals (= allometry-free shape), for Procrustes shape coordinates (= shape), and for Procrustes shape coordinates and log centroid size (= form). When *Κ* > 1, the phylogenetic signal is greater than what is expected under a Brownian motion model (i.e., variance is greater among clades), and lower if *Κ* < 1 (i.e., variance is greater within clades). *K* = 1 corresponds to a morphology that follows a Brownian motion model (i.e., perfect drift). We performed permutations of the shape data among the tips of the phylogeny with 1,000 iterations, to test for statistical significance against the null hypothesis of no pattern of similarity among relatives (i.e., no phylogenetic signal). We conducted the evaluation of the phylogenetic signal using the “physignal” function of the *geomorph* package (Adams *et al*., 2021; Baken *et al*., 2021). The reference tree (Appendix S5) was the consensus chronogram tree (branch length proportional to absolute time) for the Bayesian phylogeny of the 25 modern species, based on the GenBank dataset and downloaded from the version 2 of 10kTrees website (Arnold *et al*., 2010; https://10ktrees.nunn-lab.org/index.html).

## Ethical note

The fossil specimen was found during paleontological excavations in Greece. We conducted the excavations according to local regulations, with due authorization from all relevant parties. All primates included in the comparative sample were dry skulls from historical collections housed in public museums or universities. Therefore, our study did not imply specific issues regarding animal welfare. For all specimens, we had the relevant authorization (depending on the kind of data; see below) to perform microCT-scanning, to use microCT-scans for the virtual extraction, and/or to use 3D mesh surfaces of the labyrinth. The authors declare no conflict of interest.

### Data availability

All data generated or analysed during this study are included in this published article and its supplementary information files.

## Results

### Comparative anatomy

**Fig. 2:**
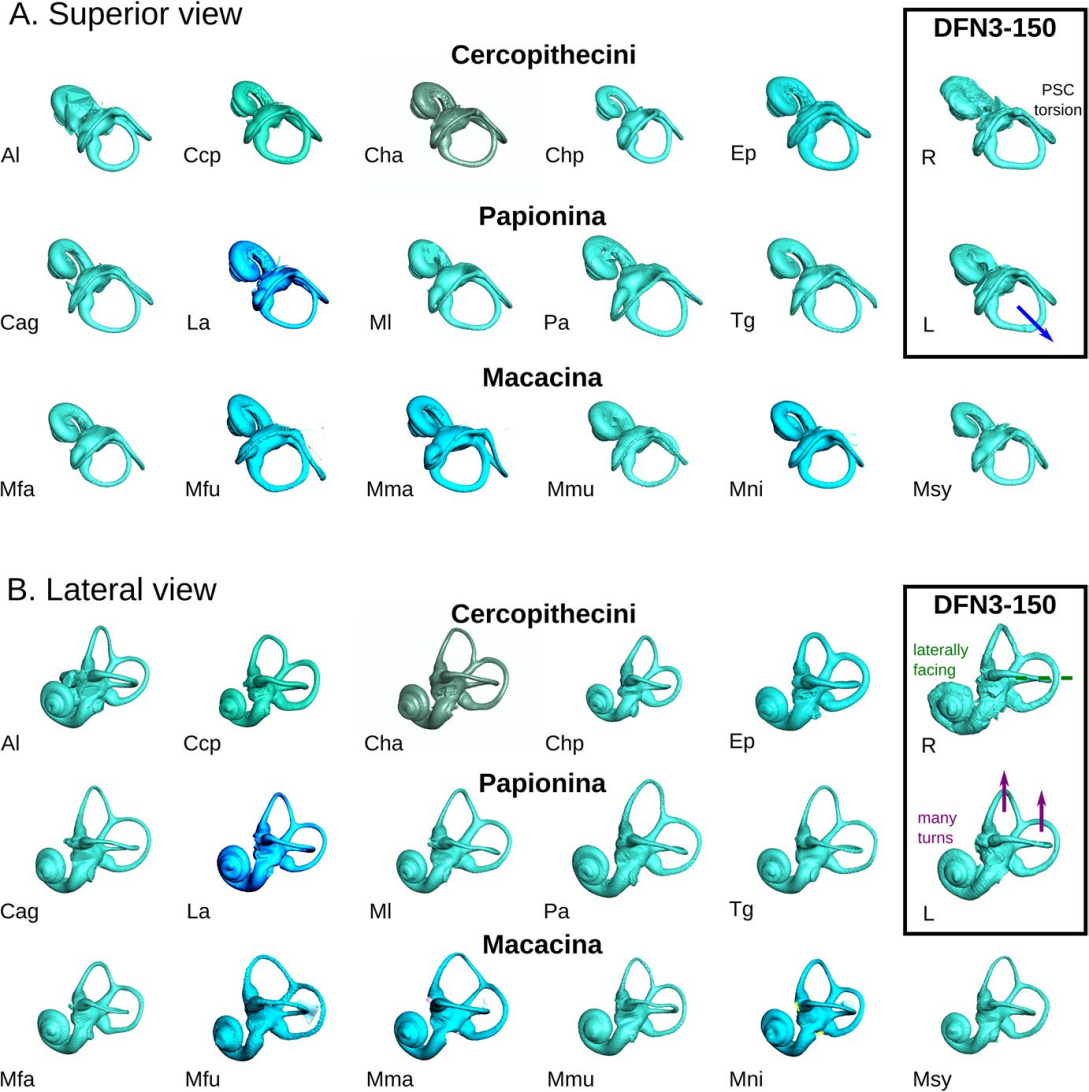
Bony labyrinth of extant species, compared to the two labyrinth (*R*, right; *L*, left) of the fossil specimen *Paradolichopithecus aff. arvernensis* LGPUT DFN3-150. The bony labyrinth is visualised as from the left side (right side mirrored), in orthographic view. The anatomical features that the fossil shares with both Cercopithecini and Papionina (cochlea with many turns, superior projection of the ASC and PSC relative to the common crus) are highlighted in *purple*, with Papionina only (posterolateral projection of the LSC) in *blue*, and with Macacina (laterally facing cochlea, superior branching of the LSC) in *green*. The torsion pattern of the PSC is unique to the fossil taxon. **a** Superior view. **b** Lateral view. (*Top*) Cercopithecini: *Al*, *Allochrocebus lhoesti* (83–006-M153); *Ccp*, *Cercopithecus cephus* (MHNT-OST-AC-515); *Cha*, *Chlorocebus aethiops* (MHNT-OST-AC-523); *Chp*, *Chlorocebus pygerythrus* (MNHN-1972–302); *Ep*, *Erythrocebus patas* (MRAC-8452). (*Middle*) Papionina: *Cag*, *Cercocebus agilis* (AMNH-M-52635); *La*, *Lophocebus albigena* (MRAC-37572); *Ml*, *Mandrillus leucophaeus* (MNHN-2002–105); *Pa*, *Papio anubis* (AMNH-M-52668); *Tg*, *Theropithecus gelada* (MNHN-1969–449. (*Bottom*) Macacina: *Mfa*, *Macaca fascicularis* (MCZ-12758); *Mfu*, *Macaca fuscata* (AMNH-M-35640); *Mma*, *Macaca maura* (AMNH-M-90159); *Mmu*, *Macaca mulatta* (MCZ-61414); *Mni*, *Macaca nigra* (AMNH-M-196409); *Msy*, *Macaca sylvanus* (NMHN-M-476780)

Because of sediment filling, the reconstruction of the right labyrinth of the fossil LGPUT DFN3-150 is much more rugged than the left one, and little parts of the basal turn of the cochlea are missing (Fig. 2, Appendix S4-1 and S4-2). Compared to the left side, it is slightly distorted, with a somewhat flattened basal turn of the cochlea, and a slight elongation along the anteromedial–posterolateral axis. The left labyrinth is not distorted.

*Paradolichopithecus* labyrinth morphology does not exactly match that of any extant species, but it is most similar to *Erythrocebus patas*, a Cercopithecini, and *Mandrillus leucophaeus*, a Papionina (Fig. 2). It shares with both Cercopithecini and Papionina i) a cochlea with more than three turns, and ii) a superior projection of the anterior and posterior semicircular canals (ASC and PSC, respectively) relative to the common crus, even if the latter canal is relatively less projected in Papionina because of their longer common crus. The specimen LGPUT DFN3-150 also shares with Papionina a posterolaterally projected lateral semicircular canal (LSC), even when taking into account the potential distortion of the fossil. However, some similarities can also be found with Macacina, such as the more laterally facing cochlea, and the branching of the posterior part of the LSC on the vestibule that is more superior relative to the PSC (a feature also observed in some Cercopithecini). Finally, some features are specific to *Paradolichopithecus* labyrinth, such as its unique torsion pattern for the PSC.

### Size variation and allometry

When only extant species are considered (*n* = 71, excluding *Macaca* sp. and observations with undetermined sex), there is a statistically significant positive correlation between labyrinth centroid size and body mass (*R*^2^ = 0.4765; F_1,69_ = 62.8; *p* < 0.005). Procrustes shape coordinates are also significantly associated with body mass (*R*^2^ = 0.0272; F_1,69_ = 1.9287; *p* = 0.0078). When the additive effect of body mass and labyrinth centroid size is evaluated, we find a significant association of both variables with Procrustes shape coordinates (*p* = 0.0067 for body mass and *p* = 0.0033 for centroid size). For a model with an interaction term between body mass and labyrinth centroid size, we still find significant associations with shape coordinates (*p* = 0.0062 and *p* = 0.0028, respectively), with a significant interaction effect (*p* = 0.0169).

**Fig. 3:**
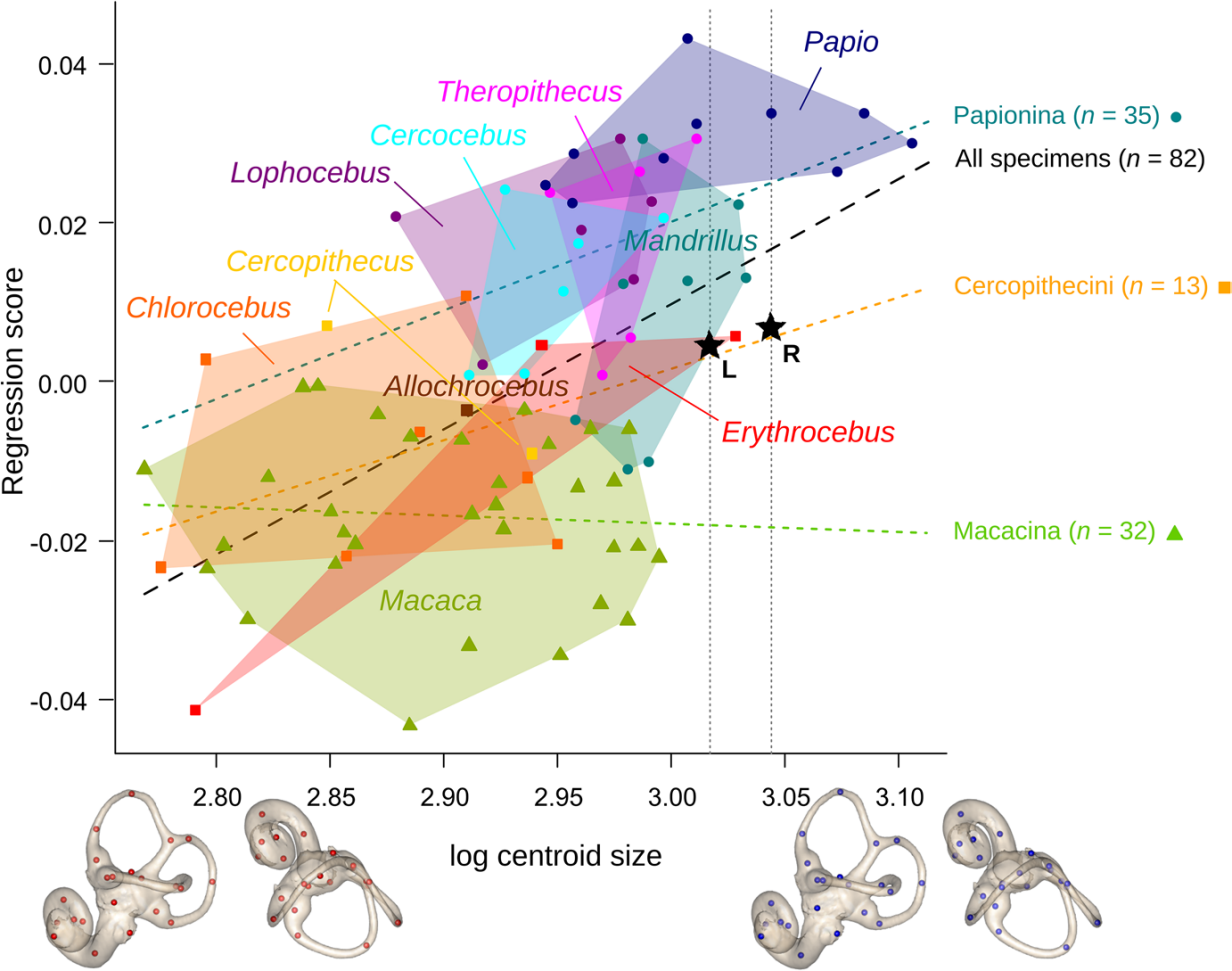
Allometric trend for the labyrinth in Cercopithecinae. The labyrinth centroid sizes (in mm) are shown in logarithmic scale. The fossil specimen LGPUT DFN3-150 is represented by *stars* for the left (*L*) and right (*R*) sides. The *squares* correspond to Cercopithecini, the *circles* correspond to Papionina, and the *triangles* correspond to Macacina. *Convex hulls* delineate genera. The predicted shape configurations at minimum and maximum sizes are represented as warped surfaces (orthographic projections of the left surface – *left*, lateral view; *right*, superior view). The regression lines of the regression score on log centroid size are plotted for the whole sample (*black line*) and for the different clades: Cercopithecini (*orange*), Papionina (*turquoise*) and Macacina (*green*). The *vertical lines* correspond to centroid size for each side of the fossil LGPUT DFN3-150

Papionina tend to have a larger bony labyrinth than the other Cercopithecinae, especially *Papio anubis* (Fig. 3). In contrast, Cercopithecini have a small labyrinth, except for the two *Erythrocebus* male specimens. For labyrinth size, Macacina overlap with Cercopithecini. They also completely overlap with the smallest Papionina (*Lophocebus*, *Theropithecus* and *Cerocebus*) and partially with larger Papionina (*Mandrillus* and *Papio*), except *Macaca fascicularis* and *M. nigra*, which have a smaller labyrinth.

Size has a significant effect on shape for the bony labyrinth, but the association is weak (*n* = 82, *F*_1,80_ = 1.828, multiple *R*^2^ = 0.021, *p* < 0.001). Compared to Macacina, all Papionina have i) a larger, more superiorly projected, and less twisted ASC, ii) a LSC which is more twisted, more posterolaterally projected and less anteriorly projected, and iii) a more twisted and coiled first cochlear turn (Fig. 3). Only three *Mandrillus leucophaeus* (female or unidentified sex) share some shape similarities with some macaques. Cercopithecini have intermediate shapes between Macacina and Papionina, with more similarities to the former, especially for one female *Erythrocebus patas* having extreme *Macaca*-like features. The fossil specimen (LGPUT DFN3-150; a female specimen; Kostopoulos *et al*., 2018) is characterized by high CS values, similar to *Papio*, *Theropithecus*, *Mandrillus*, and male *Erythrocebus patas*, and it has an intermediate shape, like Cercopithecini, but closer to Papionina (Fig. 3; Appendix S6: Fig. 2).

The regression lines for Cercopithecini and Papionina are roughly parallel, and their slopes have the same sign as the overall regression line (slope = 0.16), but unlike the regression slope for Macacina (Fig. 3; Appendix S6: Fig. 1). The fossil LGPUT DFN3-150 is positioned on the Cercopithecini line and close to the overall regression line, but far below the Papionina line (but three *Mandrillus* are further away), and clearly above the Macacina line beyond the 90% prediction band (Appendix S6: Fig. 1).

### Shape variation

**Fig. 4:**
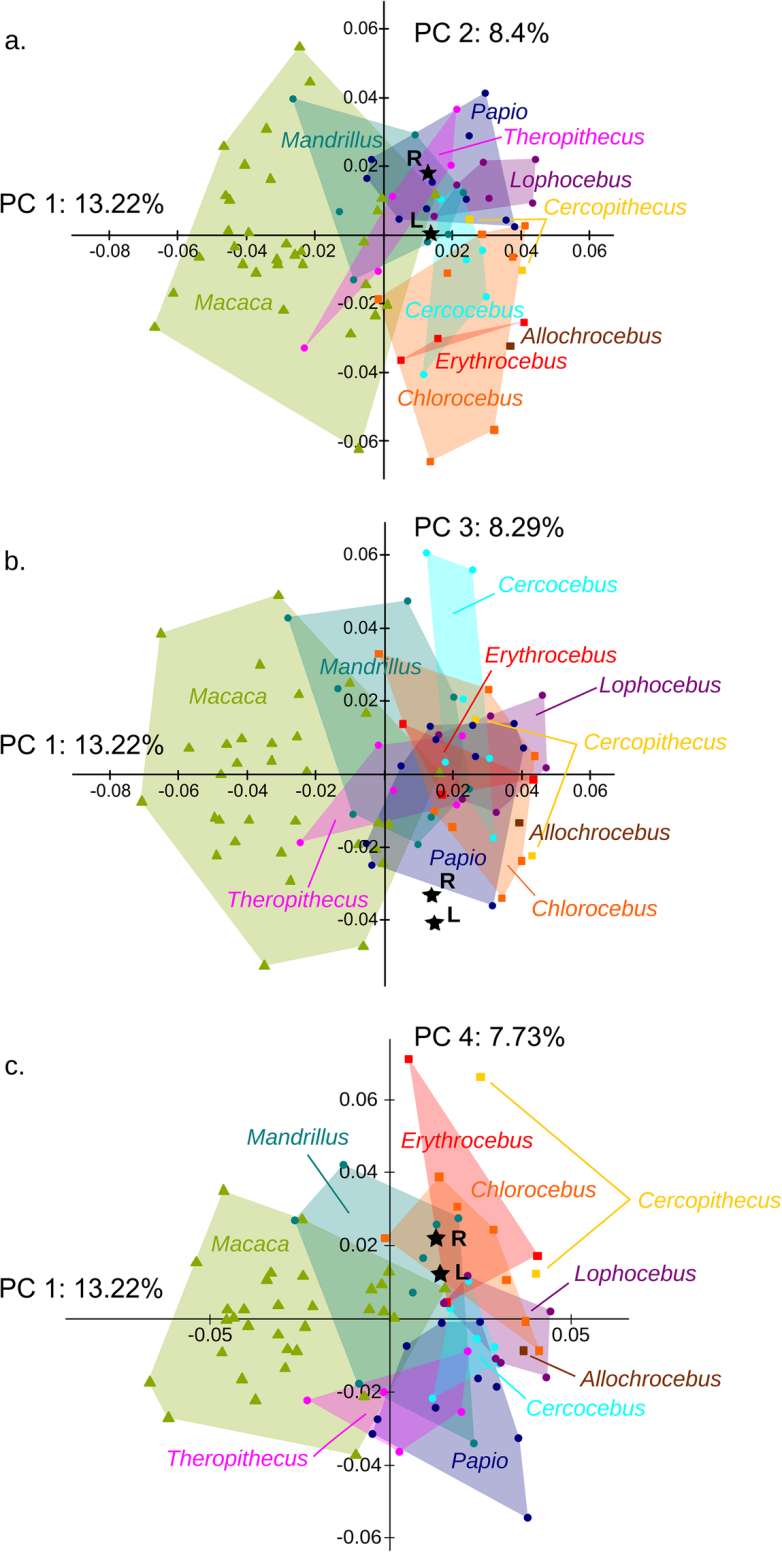
Four first principal components of the Procrustes shape coordinates of the bony labyrinth in Cercopithecinae. **a** PC2 vs PC1. **b** PC3 vs PC1. **c** PC4 vs PC1. See legend of Fig. 3 for a detailed description of symbols

Although with considerable overlap, the first four principal components of the Procrustes shape coordinates (37.6% of the total variance; see Appendix S7: Fig. 1) separate three groups: Cercopithecini, Papionina, and Macacina (Fig. 4; Appendix S7: Figs. 2, 3; Appendix S8). Despite considerable overlap, especially for *M. fascicularis*, the first principal component (PC1; 13.2% of the total variance) tends to separate Macacina (lower scores) from the two other groups, whereas the second principal component (PC2; 8.4% of the total variance) distinguishes Cercopithecini (lower scores) from Papionina. Papionina overlap with both groups. *Mandrillus*, *Theropithecus* and *Papio* overlap with Macacina along PC1, while *Cercocebus* and *Theropithecus* overlap with Cercopithecini along PC2. There is no clear trend along the third principal component (PC3; 8.3% of the total variance), except a separation between the two *Cercocebus torquatus* (high scores) and the other *Cercocebus* species and, within Papionina, a distinction between the genera *Cercocebus* and *Mandrillus* (higher scores) on one end and the other papionin genera on the other end. The fourth principal component (PC4; 7.7% of the total variance) partially separates Cercopithecini (higher scores) from certain Papionina (*Theropithecus*, *Papio*). Considering together the four first principal components, the fossil specimen has no clear affinities with any clade, but it slightly tends to group with Papionina, very close to both *Papio* and *Mandrillus* (Fig. 4; Appendix S7: Figs. 2, 3; Appendix S8).

The shape variation associated with each principal component corresponds to changes in both the cochlea and the semicircular canal system. These shape changes are different from allometric shape changes, except some similarities for PC4 such as the posterolateral projection of the LSC and the superior projection of the ASC (for a detailed description of the shape variation patterns, see Appendix S7: Fig. 4). The fossil specimen has positive PC1 scores because of the posterolateral projection of the LSC and the superior projection of the vertical semicircular canals (relative to the short common crus), a condition shared with Papionina. Its slightly positive PC2 scores are related to the laterally facing cochlea. Its strongly negative PC3 scores correspond to a less round LSC, an increased torsion of the PSC, and more superoinferiorly projected vertical semicircular canals. Finally, its slightly positive PC4 scores are due to the inferior projection of the PSC relative to the LSC.

The association between each principal component and centroid size is very weak for PC1, PC2, and PC4, and completely negligible for PC3 (correlation coefficients as low as PC1 0.21, PC2 0.15, PC3 0.02, and PC4 − 0.23). Therefore, it is not surprising to find a very similar distribution of the species for the PCA on Procrustes shape coordinates (described above and in Appendices S7 and S8) and for the PCA on the residuals of their regression on log centroid size (Appendix S9: Figs. 1 and 2).

**Fig. 5:**
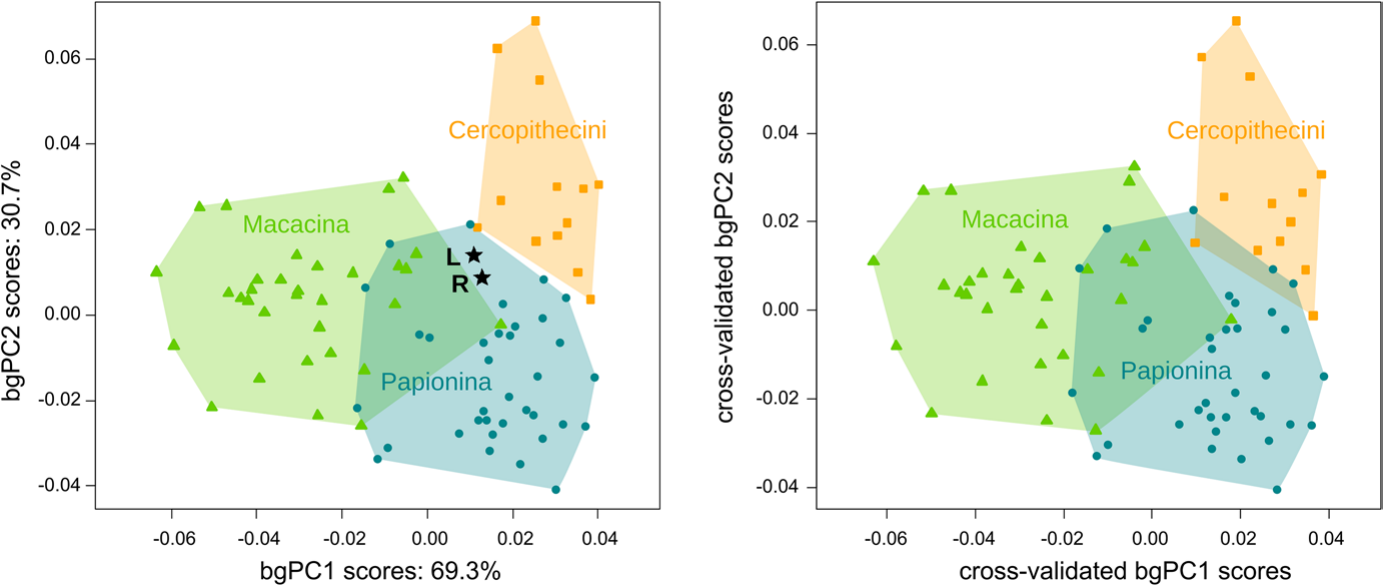
Between-group principal components (bgPC) of seven shape variables describing the bony labyrinth of extant cercopithecines. The shape variables were the first seven principal components of Procrustes shape coordinates. Convex hulls enclose each group used for the classification, and the black stars corresponds to the projection of the left (*L*) and right (*R*) labyrinths of the fossil LGPUT DFN3-150 on the space of bgPC1 and bgPC2. The *left panel* represents the scores for bgPC2 (30.7% of the between-group variance explained) vs bgPC1 (69.3% of the between-group variance explained). The *right panel* represents the cross-validated scores

For the bgPCA conducted on the first seven PCs of the Procrustes shape variables, the classification success is good, with 86.25% correct classification for the leave-one-out cross-validation (91.25% without cross-validation). With the leave-one-out cross-validation, six Papionina (8.6%) were misclassified as Macacina (three) and as Cercopithecini (three); four Macacina (12.5%) were misclassified as Papionina (two) or Cercopithecini (two); and only one Cercopithecini (7.7%) was misclassified as Papionina. There is a strict separation between Cercopithecini and Macacina along the first between-group principal component (69.3% of the explained variance between groups) and, when both bgPCs are considered, Papionina occupy an intermediate position that partially overlaps with Macacina, especially *M. fascicularis*, while being separated from Cercopithecini (Fig. 5a; Appendix S10). The bgPC scores are very similar to their cross-validated counterparts, except a limited overlap between Papionina and Cercopithecini for cross-validated scores, which means that there is no spurious group separation (Fig. 5b; Appendix S10). The fossil LGPUT DFN3-150 is located within the shape distribution of Papionina. It is outside the shape distribution of both Macacina and Cercopithecini when the two bgPCs are taken into account, but it remains close to the border of the convex hulls of both clades. Typicality probabilities do not return a clear grouping for the fossil LGPUT DFN3-150, except that it is not a Macacina. Whereas the left labyrinth is classified as a Cercopithecini, the right side is classified as a Papionina (Table II).

**Table II:** Typicality probabilities for the fossil LGPUT DFN3-150 to belong to each clade, based on the bgPCs. Probabilities are given separately for the left and right sides, with the highest probability in bold

	Cercopithecini	Macacina	Papionina
Left	**0.323**	0.043	0.211
Right	0.248	0.040	**0.354**

**Fig. 6:**
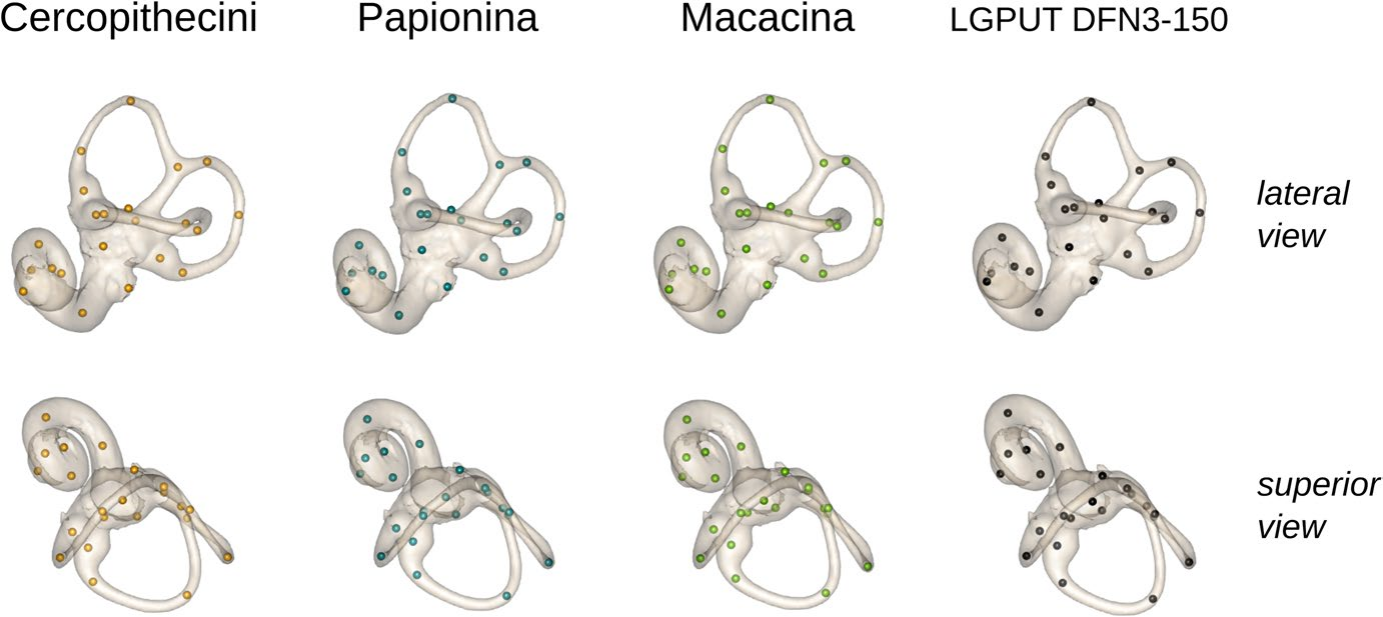
Shape configuration (average of left and right sides) of the fossil LGUT DFN3-150, compared to the average shape configurations of Cercopithecini, Papionina, and Macacina (orthographic projections of the left surface —–*top*, lateral view; *bottom*, superior view). The average shape configurations are warped surfaces obtained from the deformation of the surface of a reference specimen (specimen *Theropithecus gelada* MNHN-ZM-MO-1972-360), based on Procrustes shape coordinates

Compared to the average shape configuration of the three extant clades (Fig. 6), the bony labyrinth of *Paradolichopithecus* aff. *arvernesis* is elongated in the anteromedial–posterolateral axis, with smaller and less round semicircular canals, a LSC that branches more superiorly on the common crus, and a more horizontal cochlear axis. The posterolateral projection of the LSC is common between *Paradolichopithecus* and the average Papionina. Relative to the average Papionina and Cercopithecini, the fossil shares some similarities with the average Macacina, such as a short common crus, a less twisted LSC, and a less superiorly projected ASC. However, contrary to *Paradolichopithecus*, the common crus is shifted superiorly and the superior part of the PSC is projected anteriorly in the average Macacina.

### Clustering analyses

**Fig. 7:**
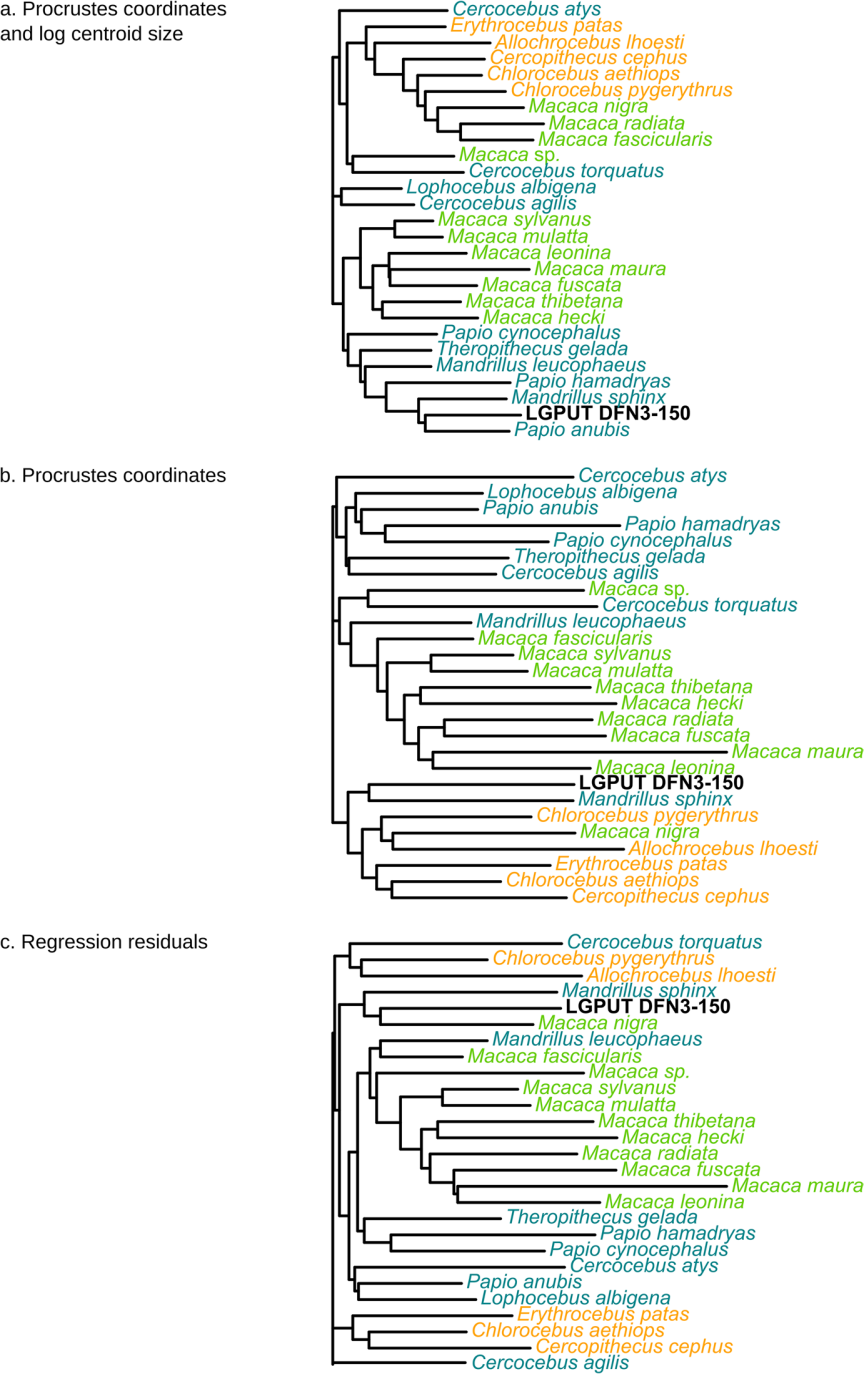
Dendrograms resulting from NJ cluster analyses based on the Euclidean distances among species computed for (**a**) Procrustes shape coordinates and log centroid size; (**b**) Procrustes shape coordinates; (**c**) Residuals from the regression of Procrustes shape coordinates on log centroid size. *Orange*, Cercopithecini; *green*, Macacina; *turquoise*, Papionina

Dendrograms reconstructed from neighbour-joining clustering algorithm differ depending on the way size variation is included. When log centroid size is included, the larger Papionina (*Papio*, *Theropithecus*, *Mandrillus*) cluster together, and the fossil falls within this group, closest to *Papio anubis* and *Mandrillus sphinx* (Fig. 7a). A cluster of seven larger *Macaca* forms the nearest group of the cluster of larger Papionina. All Cercopithecini cluster together, with a group of three small *Macaca* species branching inside. The smaller Papionina (*Cercocebus*, *Lophocebus*) branch more basally relative to these clusters. Labyrinth centroid size is clearly the main driver of the clusterings in this case.

For the analysis based on Procrustes shape coordinates only, all Macacina cluster together, except for a *Macaca* sp. and the *M. nigra* specimen (Fig. 7b). The latter falls inside another cluster made by all Cercopithecini. Most Papionina cluster together, except *Cercocebus torquatus* and the two species of *Mandrillus*. *Mandrillus sphinx* with the fossil taxon forms the nearest group of Cercopithecini, whereas *Mandrillus leucophaeus* and *Cercocebus torquatus* cluster with the Macacina group.

7The analysis based on regression residuals shows many similarities with the analysis based on Procrustes shape coordinates with regard to the Macacina cluster (Fig. 7c). However, Papionina and Cercopithecini are mixed in several small clades that all branch basally relative to Macacina. As in other clustering analyses, the fossil taxon groups with *Mandrillus sphinx*, together with *Macaca nigra*, but outside the Macacina cluster. For all analyses, the pattern of clustering within each subtribe (Cercopithecini, Macacina and Papionina) is not consistent with the molecular phylogeny (Fig. 7a–c).

### Phylogenetic signal

When all extant cercopithecines are considered, we get a weak phylogenetic signal (0.3 < *K*_mult_ < 0.4), with closely related taxa having less similar morphologies than the expectation under a Brownian motion model: shape and size variance is larger within clades than among clades (Table III). The phylogenetic signal is slightly weaker in centroid size, compared to Procrustes shape coordinates or regression residuals. We get very similar results when the analyses are conducted for extant Papionini only (Table III). *P*-values are below the 5% threshold as long as Procrustes shape coordinates are taken into account (except for Papionini when log centroid size is considered together with the coordinates), but not for centroid size alone. However, interpreting these results is tricky, because the null hypothesis of no pattern of similarity among relatives is very unlikely to be true in any biological organism.

**Table III:** Phylogenetic signal in the morphology of the bony labyrinth for extant Cercopithecinae and for the tribe Papionini. The star (*) indicates a statistically significant signal *(p* < 0.05)

	Cercopithecinae		Papionini	
	*K* or *K*_mult_	*P*	*K* or *K*_mult_	*P*
Centroid size	0.324	0.382	0.308	0.538
Regression residuals	0.369	0.005*	0.369	0.044*
Procrustes shape coordinates	0.380	0.001*	0.383	0.013*
Procrustes shape coordinates & log centroid size	0.362	0.024*	0.356	0.143

## Discussion

Our results show a very large variation in labyrinth centroid size in extant Cercopithecinae, with a larger labyrinth for heavier primates, which means that size is an important component of morphological variation (supports H1; Table IV). However, despite some association between labyrinth shape and size, allometry is only a very small component of shape variation (*contra* H2; Table IV). Clustering analyses show that labyrinth morphology reflects phylogeny at the tribe and the subtribe levels (supports H3), but not the fine pattern at lower taxonomic levels (*contra* H3; Table IV). The latter result might be explained by the superimposition of an adaptive signal (supports H3). Centroid size alone does not bear a phylogenetic signal, and the removal of allometric change only slightly weakens the signal in labyrinth shape, which means that size might partially drive the phylogenetic signal, but only indirectly, through minor allometric shape changes (*contra* H4; Table IV).

**Table IV:** Summary of the hypotheses regarding the bony labyrinth of Cercopithecinae

Hypotheses		Validity
H1	**Size is the main component of morphological variation** Support: - There is a very large centroid size variation across species - Heavier primates have larger centroid sizes	**Yes**
H2	**Allometry is an important component of shape variation** Contradiction (allometry present, but weak): - Shape variation is associated with labyrinth size variation and body mass, but the correlations are weak - The shape differences associated with the first four PCs are not the same as the allometric shape changes, except for PC4 (only 7.7% of the total variance)	**No, only little**
H3	**Morphology reflects both phylogeny and ecology** Support for phylogeny (at the subtribe level): - The rate of successful classification is good for the bgPCA - Clustering analyses on Procrustes shape coordinates roughly separate Macacina, Papionina and Cercopithecini - There is a phylogenetic signal in labyrinth shape - When a larger taxonomic range is considered (subfamily Cercopithecinae vs. tribe Papionini), the phylogenetic signal is slightly stronger for centroid size Contradiction with phylogeny (fine phylogenetic pattern): - Clustering analyses perform poorly for the fine phylogenetic patterning within each subtribe - The phylogenetic signal in labyrinth shape is weak = > adaptive signal?	**Probably yes**
H4	**The phylogenetic signal in the bony labyrinth morphology is mainly driven by size** Support (effect of size through allometry): - Species clustering in subtribes is less consistent when centroid size is regressed out - The phylogenetic signal is weaker when centroid size is regressed out - All PCs showing some separation between clades are weakly correlated with centroid size Contradiction (no direct effect of size): - The phylogenetic signal is weaker for centroid size, compared to Procrustes shape coordinates	**No, only weakly**

Because allometry partially drives the phylogenetic signal in labyrinth shape variation in Cercopithecinae, without being its main component, we should not regress out the size component to infer the affinities of the fossil specimen. As predicted for *Paradolichopithecus*, we found a large bony labyrinth, and many shape similarities with the large Papionina, even if it also has some affinities with other cercopithecines, especially Cercopithecini. Our findings on the bony labyrinth morphology do not support closest relationships with Macacina. However, it is difficult to decide whether *Paradolichopithecus* was a Papionina retaining some ancestral traits common to most Cercopithecinae, or a stem Papionini with closer affinities with Papionina.

As has been stressed by previous authors (e.g., Jablonski, 2002; Szalay & Delson, 1979) *Procynocephalus* and *Paradolichopithecus* show a mixture of outer cranial morphometric features between the mostly African *Papio* and the predominantly Eurasian *Macaca* (both extant taxa used in these studies as proxies of the morphological and phylogenetic divergences between the two main lineages of Papionini). Several studies (e.g., Jablonski, 2002; Nishimura *et al*., 2014; O’Shea *et al*., 2016; Rae, 2008; Szalay & Delson, 1979; Takai *et al*., 2008) suggest that external cranial morphology and inner nasal architecture of *Paradolichopithecus* exclude it from Papionina and place it closer to the crown lineage of *Macaca*. Resonant exceptions are the studies of Maschenko (1994, 2005), who directly refers *Paradolichopithecus* from Kuruksay (Tajikistan) to the genus *Papio*. Similarly to outer cranial features, the bony labyrinth of *Paradolichopithecus* shares anatomical features with both Macacina and Papionina. Some characters shared with Papionina might be plesiomorphic, like the superior projection of the vertical semicircular canals and the high number of turns of the cochlea, which are also found in Cercopithecini. However, the overall anatomy of the bony labyrinth points to more affinities with Papionina, particularly with the genus *Mandrillus*.

The size similarities between the labyrinths of *Papio*, *Theropithecus*, *Mandrillus*, the male *Erythrocebus patas*, and the fossil *Paradolichopithecus* from Dafnero-3 clearly reflect the fact that they are all large cercopithecines (Fleagle, 1999; Kostopoulos *et al*., 2018). Within each Papionini subtribe, species with larger labyrinth size (*T. gelada*, *Papio* sp., *Mandrillus* sp. in Papionina, and *M. mulatta*, *M. sylvanus* in Macacina) are also those with less arboreal behavior, whereas species with the smallest labyrinth sizes (*L. albigena*, *M. fascicularis*) tend to be arboreal climbers — a trend probably linked to differences in body mass. The large labyrinth size of *Paradolichopithecus* supports post-cranial evidence of terrestrial behavior (Sondaar *et al*., 2006; Ting *et al*., 2004). The regression score for *Paradolichopithecus* corresponds to a large Cercopithecini, but it is not consistent with the extrapolation of shape for a large macaque. Therefore, the shape similarities between the fossil taxa and Macacina are more likely to reflect the retaining of several shape features that would represent the ancestral pattern for all Papionini and even all Cercopithecinae (as these features are also found in Cercopithecini), rather than the consequence of allometric shape changes in large Macacina.

The separation of each species is quite efficient when the shape of the whole labyrinth is used, contrary to previous studies based only on the shape of the semicircular canals, in which more overlap was observed (Beaudet *et al*., 2016). When considering the four first components of Procrustes shape coordinates, the separation between Papionini and Cercopithecini on the one hand, and between Macacina and Papionina on the other hand, is consistent with the classification of cercopithecines (Tosi *et al*., 2003). The separation between Papionina and the two other clades is partly driven by the allometric component, as it tends to be less good when this component is removed. The shape similarities between *Theropithecus* and *Lophocebus*, together with *Papio*, and the proximity of *Cercocebus* and *Mandrillus* also fit with the most recent phylogenies (Harris, 2000; Pugh & Gilbert, 2018; Tosi *et al*., 2003). These observations confirm that, even though the phylogenetic signal is weak, the labyrinth morphology as a whole provides some phylogenetic information at least at higher taxonomic levels (tribe, subtribe), as is observed for the semicircular canals alone (Beaudet *et al*., 2016; Urciuoli *et al*., 2020). The morphological affinities of *Paradolichopithecus* with Papionina regarding labyrinth shape speak in favour of closest phylogenetic relationships with this group, contrary to the classical hypothesis for a *Paradolichopithecus*–Macacina grouping. All clustering analyses show morphological affinities between *Paradolichopithecus* and *Mandrillus sphinx*; however, as these analyses tend to reflect size rather and not just phylogeny, it is still not clear whether this fossil belongs to the *Cercocebus*–*Mandrillus* clade, or to the *Papio*–*Theropithecus*–*Lophocebus* clade, or branches at a more basal position within Papionina or even Papionini.

Morphological similarities of the LGPUT DFN3-150 with Papionina are partially linked to centroid size of the bony labyrinth through allometric shape changes. This is visible in the clustering analyses, in which the dendrograms are less consistent with the known phylogeny when log centroid size is regressed out. The slightly weaker phylogenetic signal for regression residuals compared to Procrustes shape coordinates also points to a similar direction, although the difference remains small. Therefore, we can not exclude that the shape affinities of *Paradolichopithecus* with the baboon-related clade are the result of a parallel evolution for large, terrestrial species that would arise from a common allometric pattern across all cercopithecines (e.g., Nishimura *et al*., 2019). The Blomberg’s *K* and *K*_mult_ values, which are all inferior to one, show that morphological variance is accumulated within clades, which is consistent with homoplasy. Though, labyrinth morphology does not depend just on CS, but also on phylogeny independently of body size. Indeed, Cercopithecini are separated from Macacina in the allometry-free shape space (with Papionina partially overlapping both of these clades at an intermediate position). This result is consistent with two recent analyses based on two different geometric morphometric protocols (Morimoto *et al*., 2020; Urciuoli *et al*., 2020).

The discrepancy between an absent or very weak signal for the phylogenetic and clustering analyses of allometry-free shape, and the good classification rates for the between-group principal component analysis, might be explained by two factors. First, the clustering analysis is based on Euclidean distances, which correspond to one variable summarizing many morphological traits. Therefore, the absence of clusters exactly similar to the molecular phylogeny does not necessarily mean that morphology does not reflect phylogeny. It only implies that the phylogenetic signal is not captured by the Euclidean distance, *taken alone*. Second, the bgPCA separates groups at high taxonomic levels (tribe, subtribe), whereas the phylogenetic and clustering analyses take into account the species level. We can interpret this as follows: when allometric effects are removed, labyrinth shape reflects phylogenetic patterns at relatively high taxonomic levels (subtribe or above), but not necessarily at lower levels, which is consistent with recent findings for anthropoids (Beaudet *et al*., 2016; del Rio *et al*., 2021; Morimoto *et al*., 2020; Urciuoli *et al*., 2020). As a consequence, the position of the fossil in the allometry-free shape space is likely to reflect a phylogenetic proximity with Papionina as a group, but it cannot be used to infer its affinities with any particular extant species within this group.

Because our results demonstrate that the correlation between labyrinth shape (not size) and body mass is only weak in Cercopithecinae, we would recommend to relying more on this feature to infer phylogeny at a large taxonomical scale, rather than the outer cranial features which are known to be highly correlated with body mass and ecology. However at lower taxonomical scales, both types of evidence might be as useful, since labyrinth morphology does not seem to perform particularly well to infer the fine phylogenetic pattern. This could be explained by the fact that labyrinth morphology is also associated with cranial base shape (Jeffery & Spoor, 2004; Le Maître, 2019; Spoor & Zonneveld, 1998), which depends on body mass and ecology.

The rough morphological resemblance of some young *Paradolichopithecus* individuals, such as LGPUT DFN3-150, with *Macaca* could be justified by the similar pre-adult ontogenetic trajectories followed by male and female macaques, mandrills, mangabeys, and baboons (Leigh, 2006; Mottura & Gentili, 2006). Taking into account all these elements, together with the findings of the present study, a hypothesis of *Paradolichopithecus –* Papionina relationship appears more supported than the traditional *Paradolichopithecus –* Macacina one. An alternative hypothesis would be that *Paradolichopithecus* was a basal Papionini, closer to Papionina relative to Macacina, sharing common shape features with both groups because of common ancestry and convergent allometric pattern for all Cercopithecinae. This hypothesis might be ruled out with further analyses conducted on basal African Papionini, which is beyond the scope of the present paper.

## Concluding remarks

Our results confirm that, even using a small number of landmarks, the morphology of the bony labyrinth can be used to infer phylogenetic relationships of primate species. However, as highlighted in other studies, it is reliable only at taxonomic levels higher than the family. As in other primate groups, we found an important size variation, which is the main driver of the phylogenetic signal in labyrinth morphology. Surprisingly, despite this large size variation, allometry is only a minor component of labyrinth shape variation in Cercopithecinae. This is a large aspect for the reconstruction of phylogenetic affinities of other fossil papionins, as the main issue in this group is generally to disentangle the phylogenetic and the allometric components in morphological features. Our findings do not provide a clear answer to the taxonomic classification of *Paradolichopithecus*, though the traditional hypothesis of a *Paradolichopithecus* –Macacina relationship appears to be the least supported. In any case, the results of the present study reveal that the evolutionary history of Eurasian late Neogene–Quaternary cercopithecids might be more complicated than previously thought, and point to the need for a fresh in-depth phylogenetic investigation.

## Supplementary Information

Below is the link to the electronic supplementary material.

ESM 1 (DOCX 21 kb)

ESM 2 (DOCX 10 kb)

ESM 3 (CSV 91 kb)

ESM 4 (PLY 11413 kb)

ESM 5 (PLY 11947 kb)

ESM 6 (NEX 1 kb)

ESM 7 (DOC 65 kb)

ESM 8 (DOCX 4308 kb)

ESM 9 (HTML 1144 kb)

ESM 10 (DOCX 79 kb)

ESM 11 (DOC 78 kb)
