# Supplementary material for: Morphology of the Bony Labyrinth Supports the Affinities of Paradolichopithecus with the Papionina
Source: Int J Primatol. 2022 Sep 20;44(1):209–36. doi: 10.1007/s10764-022-00329-4 (PMC9931825; doi:10.1007/s10764-022-00329-4)
Supplement: Supplementary file 1 — (DOCX 21 kb) [file 10764_2022_329_MOESM1_ESM.docx]

**S1 Table.** Comparative sample used for the study of the bony labyrinth.

| Species | ID | Sex^a^ | Age^b^ | Institution^c^ | HR-µCT **facility**^d^ | Voxel size (µm) ^e^ | Reference^f^  ARK/DOI (**article** / specimen) |
| --- | --- | --- | --- | --- | --- | --- | --- |
|  |  |  |  |  |  |  |  |
| **Fossil** (n = 1) |  |  |  |  |  |  |  |
| *Paradolichopithecus* aff. *arvernensis* (n = 1) | LGPUT-DFN3-150 | F | JUV | LGPUT | PLATINA | 69.9 | 10.1016/j.jhevol.2018.02.012 |
|  |  |  |  |  |  |  |  |
| **Cercopithecini** (n = 13) |  |  |  |  |  |  |  |
| *Allochrocebus lhoesti* (n = 1) | MRAC-83-006-M153 | M | SAD | RMCA | PLATINA | 19.0 |  |
| *Cercopithecus cephus* (n = 2) | MHNT-OST-AC-515 | F | JUV | MHNT | FERMaT | 33.0 | 10.18563/m3.sf.489 |
|  | MHNT-OST-AC-533 | M | AD | MHNT | FERMaT | 33.0 | 10.18563/m3.sf.493 |
| *Chlorocebus aethiops* (n = 4) | MHNT-OST-AC-508 | NA | AD | MHNT | FERMaT | 33.0 | 10.18563/m3.sf.488 |
|  | MHNT-OST-AC-523 | M | SAD | MHNT | FERMaT | 33.0 | 10.18563/m3.sf.491 |
|  | MHNT-OST-AC-540 | M | SAD | MHNT | FERMaT | 33.0 | 10.18563/m3.sf.494 |
|  | MNHN-ZM-MO-1972-328 | F | AD | MNHN | PLATINA | 13.0 |  |
| *Chlorocebus pygerythrus* (n = 3) | MNHN-ZM-MO-1972-302B | F | JUV | MNHN | PLATINA | 36.0 |  |
|  | MRAC-37477 | M | SAD | RMCA | FERMaT | 33.0 | 10.18563/m3.sf.481 |
|  | MRAC-37478 | F | AD | RMCA | FERMaT | 33.0 | 10.18563/m3.sf.482 |
| *Erythrocebus patas* (n = 3) | MHNT-OST-2002-26 | F | SAD | MHNT | FERMaT | 33.0 | 10.18563/m3.sf.484 |
|  | MRAC-34851 | M | JUV | RMCA | PLATINA | 16.8 |  |
|  | MRAC-8452 | M | SAD | RMCA | FERMaT | 33.0 | 10.18563/m3.sf.476 |
|  |  |  |  |  |  |  |  |
|  |  |  |  |  |  |  |  |
|  |  |  |  |  |  |  |  |
|  |  |  |  |  |  |  |  |
| **Papionina** (n = 35) |  |  |  |  |  |  |  |
| *Cercocebus agilis* (n = 3) | AMNH-M-52635 | F | AD | AMNH | MIF | 63.2 |  |
|  | AMNH-M-52641 | M | AD | AMNH | MIF | 68.1 |  |
|  | MRAC-14337 | F | SAD | RMCA | PLATINA | 19.9 |  |
| *Cercocebus atys* (n = 1) | MRAC-28998 | M | SAD | RMCA | FERMaT | 33.0 | 10.18563/m3.sf.480 |
| *Cercocebus torquatus* (n = 2) | MRAC-73-018-M359 | F | JUV | RMCA | FERMaT | 33.0 | 10.18563/m3.sf.496 |
|  | MRAC-73-018-M389 | M | SAD | RMCA | FERMaT | 33.0 | 10.18563/m3.sf.468 |
| *Lophocebus albigena* (n = 6) | AMNH-M-52596 | F | AD | AMNH | MIF | 65.0 |  |
|  | MCZ-6209 | F | AD | MCZ | CNS | 89.7 | 10.17602/M2/M2623 |
|  | MCZ-14725 | F | AD | MCZ | CNS | 89.7 | 10.17602/M2/M2626 |
|  | MNHN-ZM-MO-1886-123 | M | AD | MNHN |  | 66.0 | * |
|  | MRAC-37572 | M | SAD | RMCA | FERMaT | 33.0 | 10.18563/m3.sf.483 |
|  | MRAC-73-029-M109 | F | SAD | RMCA | FERMaT | 33.0 | 10.18563/m3.sf.471 |
| *Mandrillus leucophaeus* (n = 6) | MCZ-19986 | M | AD | MCZ | CNS | 111.1 | 10.17602/M2/M3059 |
|  | MNHN-ZM-MO-1893-269 | NA | JUV | MNHN | PLATINA | 17.1 |  |
|  | MNHN-ZM-MO-2002-105 | M | JUV | MNHN | PLATINA | 55.4 |  |
|  | MRAC-28425 | NA | JUV | RMCA | FERMaT | 33.0 | 10.18563/m3.sf.479 |
|  | MRAC-73-029-M105 | M | JUV | RMCA | FERMaT | 33.0 | 10.18563/m3.sf.469 |
|  | MRAC-73-029-M106 | F | AD | RMCA | FERMaT | 33.0 | 10.18563/m3.sf.470 |
| *Mandrillus sphinx* (n = 2) | MHNT-OST-AC-488 | M | AD | MHNT | FERMaT | 33.0 | 10.18563/m3.sf.485 |
|  | MHNT-OST-AC-543 | M | SAD | MHNT | FERMaT | 33.0 | 10.18563/m3.sf.495 |
| *Papio anubis* (n = 7) | AMNH-M-52668 | F | AD | AMNH | MIF | 80.1 |  |
|  | AMNH-M-52674 | F | AD | AMNH | MIF | 61.1 |  |
|  | MNHN-ZM-MO-1973-116 | F | SAD | MNHN | PLATINA | 61.8 |  |
|  | MNHN-ZM-MO-1973-117 | F | AD | MNHN | PLATINA | 18.8 |  |
|  | MNHN-ZM-MO-1996-2508 | F | SAD | MNHN |  | 93.0 | * |
|  | MRAC-80-044-M101 | NA | JUV | RMCA | PLATINA | 53.6 | 10.1016/j.jhevol.2018.02.012 |
|  | MRAC-90-042-M226 | F | JUV | RMCA | PLATINA | 25.0 |  |
| *Papio cynocephalus* (n = 2) | MRAC-17979 | M | AD | RMCA | FERMaT | 33.0 | 10.18563/m3.sf.477 |
|  | MRAC-3503 | F | SAD | RMCA | FERMaT | 33.0 | 10.18563/m3.sf.475 |
| *Papio hamadryas* (n = 1) | MRAC-97-020-M004 | M | AD | RMCA | PLATINA | 26.8 |  |
| *Theropithecus gelada* (n = 5) | AMNH-M-238034 | F | AD | AMNH | MIF | 120.3 |  |
|  | MNHN-ZM-MO-1969-449 | M | SAD | MNHN | PLATINA | 74.0 |  |
|  | MNHN-ZM-MO-1969-450 | M | AD | MNHN | PLATINA | 21.9 |  |
|  | MNHN-ZM-MO-1969-451 | F | AD | MNHN |  | 76.0 | * |
|  | MNHN-ZM-MO-1972-360 | M | AD | MNHN |  | 96.0 | * |
|  |  |  |  |  |  |  |  |
| **Macacina** (n = 32) |  |  |  |  |  |  |  |
| *Macaca fascicularis* (n = 11) | MCZ-12758 | F | AD | MCZ | CNS | 61.6 | 10.17602/M2/M3028 |
|  | MCZ-22277 | NA | AD | MCZ | CNS | 61.6 | 10.17602/M2/M3029 |
|  | MCZ-23812 | M | AD | MCZ | CNS | 61.6 | 10.17602/M2/M3030 |
|  | MCZ-23813 | F | AD | MCZ | CNS | 61.6 | 10.17602/M2/M3031 |
|  | MCZ-35058 | M | AD | MCZ | CNS | 61.6 | 10.17602/M2/M3032 |
|  | MCZ-35765 | F | AD | MCZ | CNS | 61.6 | 10.17602/M2/M3033 |
|  | MCZ-35937 | F | AD | MCZ | CNS | 61.6 | 10.17602/M2/M3034 |
|  | MCZ-35938 | F | AD | MCZ | CNS | 61.6 | 10.17602/M2/M3035 |
|  | MCZ-36030 | F | AD | MCZ | CNS | 61.6 | 10.17602/M2/M3036 |
|  | MCZ-41167 | M | AD | MCZ | CNS | 90.9 | 10.17602/M2/M3038 |
|  | MCZ-BOM-8461 | NA | AD | MCZ | CNS | 61.6 | 10.17602/M2/M3027 |
|  |  |  |  |  |  |  |  |
| *Macaca fuscata* (n = 3) | AMNH-M-35640 | NA | AD | AMNH | MIF | 117.3 | ark:/87602m4/M12489 |
|  | MCZ-37709 | M | AD | MCZ | CNS | 90.8 | 10.17602/M2/M3043 |
|  | MCZ-61273 | M | AD | MCZ | CNS | 90.8 | 10.17602/M2/M3045 |
| *Macaca hecki* (n = 2) | AMNH-M-152896 | M | AD | AMNH | MIF | 113.5 | ark:/87602m4/M19974 |
|  | AMNH-M-196405 | M | AD | AMNH | MIF | 123.3 | ark:/87602m4/M20103 |
| *Macaca leonina* (n = 1) | AMNH-11090 | M | AD | AMNH | MIF | 114.0 | ark:/87602m4/M13787 |
| *Macaca maura* (n = 1) | AMNH-M-90159 | F | AD | AMNH | MIF | 63.4 | ark:/87602m4/M12489 |
| *Macaca mulatta* (n = 4) | MCZ-26475 | M | AD | MCZ | CNS | 90.8 | 10.17602/M2/M3052 |
|  | MCZ-30384 | F | AD | MCZ | CNS | 90.8 | 10.17602/M2/M3049 |
|  | MCZ-61414 | M | AD | MCZ | CNS | 90.8 | 10.17602/M2/M3053 |
|  | MHNT-OST-AC-492 | F | AD | MHNT | FERMaT | 33.0 | 10.18563/m3.sf.486 |
| *Macaca nigra* (n = 1) | AMNH-M-196409 | M | AD | AMNH | MIF | 64.2 | ark:/87602m4/M20115 |
| *Macaca radiata* (n = 1) | AMNH-M-163078 | M | AD | AMNH | MIF | 98.8 | ark:/87602m4/M20068 |
| *Macaca* sp. (n = 1) | MHNT-OST-AC-532 | M | AD | MHNT | FERMaT | 33.0 | 10.18563/m3.sf.492 |
| *Macaca sylvanus* (n = 5) | MCZ-BOM-7072 | F | AD | MCZ | CNS | 87.7 | 10.17602/M2/M3055 |
|  | MCZ-BOM-7098 | M | AD | MCZ | CNS | 90.8 | 10.17602/M2/M3057 |
|  | MHNT-OST-AC-493 | M | AD | MHNT | FERMaT | 33.0 | 10.18563/m3.sf.487 |
|  | NMNH-M-476780 | M | AD | NMNH | MIF | 65.6 | ark:/87602m4/M20357 |
|  | UP-PRI-4-007 | M | AD | PALEVOPRIM | PLATINA | 53.6 | 10.1016/j.jhevol.2018.02.012 |
| *Macaca thibetana* (n = 2) | AMNH-129 | NA | AD | AMNH | MIF | 100.5 | ark:/87602m4/M13425 |
|  | AMNH-M-84472 | M | AD | AMNH | MIF | 74.3 | ark:/87602m4/M13766 |
|  |  |  |  |  |  |  |  |
|  |  |  |  |  |  |  |  |

^a^ Sex: F, female; M, male; NA, not available.

^b^ Age: AD, adult; JUV, juvenile; SAD, subadult.

^c^ Institutions: AMNH, American Museum of Natural History, New York, USA; LGPUT, Museum of Geology-Palaeontology-Palaeoanthropology, University of Thessaloniki, Greece; MCZ, Harvard Museum of Comparative Zoology, Cambridge, USA; MNHN, National Museum of Natural History, Paris, France; MHNT, Museum of Natural History, Toulouse, France; NMNH, Smithsonian National Museum of Natural History, Washington D.C., USA; PALEVOPRIM, Laboratory Paleontology, Evolution, Paleoecosystems, Paleoprimatology, University of Poitiers, France; RMCA, Royal Museum of Central Africa, Tervuren, Belgium; UHL, University Hospital, Leuven, Belgium; UP, University of Poitiers, Poitiers, France.

^d^ HR-µCT-scanning facilities: CNS, Harvard Center for Nanoscale Systems, Cambridge, USA; FERMaT, Fluides Energie Réacteurs Matériaux et Transferts, Toulouse, France; MIF, Microscopy and Imaging Facility of the AMNH, New York, USA; PLATINA, PLATeforme INstrumentale d’Analyse, IC2MP, Université de Poitiers, France.

^e^ The fossil cranium LGPUT DFN3-150 of *Paradolichopithecus aff. arvernensis* is housed in the Laboratory of Geology and Paleontology of the University of Thessaloniki (LGPUT). High-resolution micro-computed tomography (HR-μCT) images taken by scanning the specimen with EasyTom XL Duo µCT (using a sealed Hamamatsu microfocus x-ray source - 75W, 150 kV - and an amorphous silicon based detector Varian PaxScan 2520DX - 1536*1920 pixel matrix; 127µm pixel pitch, 16 bits, CsI conversion screen - from RX-Solutions, France) at the PLATeforme INstrumentale d’Analyse – PLATINA (IC2MP, Université de Poitiers). Beam intensity was set at 105kV and tube current at 500 µA. The LGPUT DFN3-150 cranium was acquired with 4320 projections resulting in 1771 slices of 1367*2571 pixels using a cone-beam reconstruction algorithm. The isovoxel size was set to 0.070 mm. One extant adult male cranium *Macaca* *sylvanus* (specimen UP-PRI-4-007) housed at PALEVOPRIM was also scanned with EasyTom XL Duo µCT using the same facilities. Beam intensity was set at 90kV and tube current at 390 µA. The *Macaca* *sylvanus* UP-PRI-4-007 cranium was acquired with 4320 projections resulting in 1784 slices of 1784*2988 pixels with 0.054 mm isovoxel size.

^f^ The stars correspond to a personal communication by Amélie Beaudet.
