# Supplementary material for: Morphology of the Bony Labyrinth Supports the Affinities of Paradolichopithecus with the Papionina
Source: Int J Primatol. 2022 Sep 20;44(1):209–36. doi: 10.1007/s10764-022-00329-4 (PMC9931825; doi:10.1007/s10764-022-00329-4)
Supplement: Supplementary file 2 — (DOCX 10 kb) [file 10764_2022_329_MOESM2_ESM.docx]

**S2 Table.** Definition of the 22 landmarks on the bony labyrinth (Lebrun et al., 2010). See figure 1 for visualisation.

| **#** | Name | Definition |
| --- | --- | --- |
| 1 | Helix basis | Centroid of the first turn of the cochlea |
| 2 | Helix apex | Centroid of the last turn of the cochlea |
| 3 | Helix anteromedial | Anteromedial-most point of the first turn of the cochlea |
| 4 | Helix posterolateral | Posterolateral-most point of the first turn of the cochlea |
| 5 | Helix inferior | Inferior-most point of the first turn of the cochlea |
| 6 | Helix superior | Superior-most point of the first turn of the cochlea |
| 7 | Fenestra cochlea | Centroid of the round window |
| 8 | Fenestra vestibuli | Centroid of the oval window |
| 9 | Aquaeductus vestibuli | Opening of the vestibular aqueduct in the vestibular wall |
| 10 | Crus commune apex | Bifurcation point of the common crus |
| 11 | Canalis lateralis ampulla | Centroid of the ampulla of the lateral semicircular canal |
| 12 | Canalis lateralis posteromedial | Posteromedial-most point of the lateral semicircular canal |
| 13 | Canalis lateralis posterolateral | Posterolateral-most point of the lateral semicircular canal |
| 14 | Canalis lateralis anterolateral | Anterolateral-most point of the lateral semicircular canal |
| 15 | Canalis anterior ampulla | Centroid of the ampulla of the anterior semicircular canal |
| 16 | Canalis anterior anterolateral | Anterolateral-most point of the anterior semicircular canal |
| 17 | Canalis anterior superior | Uppermost point of the anterior semicircular canal |
| 18 | Canalis anterior inferior | Inferior-most point of the anterior semicircular canal |
| 19 | Canalis posterior ampulla | Centroid of the ampulla of the posterior semicircular canal |
| 20 | Canalis posterior inferior | Inferior-most point of the posterior semicircular canal |
| 21 | Canalis posterior superior | Uppermost point of the posterior semicircular canal |
| 22 | Canalis posterior posterolateral | Posterolateral-most point of the posterior semicircular canal |
