# Supplementary material for: Morphology of the Bony Labyrinth Supports the Affinities of Paradolichopithecus with the Papionina
Source: Int J Primatol. 2022 Sep 20;44(1):209–36. doi: 10.1007/s10764-022-00329-4 (PMC9931825; doi:10.1007/s10764-022-00329-4)

**Appendix S6.** Allometric trend for the labyrinth in Cercopithecinae, visualised as a scatter plot of the regression score as a function of log centroid size. The labyrinth centroid sizes (in mm) are shown in logarithmic scale.

**Figure 1.** Visualisation of the regression lines (solid lines) for the whole sample (black) and for each clade: Cercopithecini (orange lines and squares), Papionina (turquoise lines and circles) and Macacina (green lines and triangles). The corresponding 90% confidence interval (shaded area) and the 90% prediction bands (dashed lines) are added. The fossil specimen LGPUT DFN3-150 is represented by black diamonds.


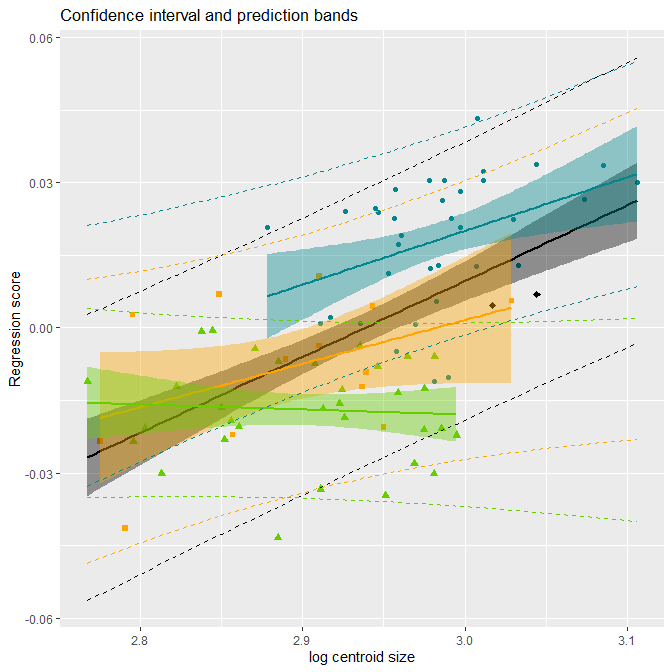


**Figure 2.** Allometric trend for the labyrinth in Cercopithecinae. This figure is another version of Fig. 3 of the main text in which species names are displayed.

Al, *Allochrocebus lhoesti*; Cag, *Cercocebus agilis*; Cat, *Cercocebus atys*; Cce, *Cercopithecus cephus*; Cha, *Chlorocebus aethiops*; Chp, *Chlorocebus pygerythrus*; Cto, *Cercocebus torquatus*; Ep, *Erythrocebus patas*; La, *Lophocebus albigena*; Mfa, *Macaca fascicularis*; Mfu, *Macaca fuscata*; Mhe, *Macaca hecki*; Ml, *Mandrillus leucophaeus*; Mle, *Macaca leonina*; Mma, *Macaca maura*; Mmu, *Macaca mulatta*; Mni, *Macaca nigra*; Mra, *Macaca radiata*; Ms, *Mandrillus sphinx*; Msp, *Macaca* sp.; Msy, *Macaca sylvanus*; Mth, *Macaca thibetana*; Pa, *Papio anubis*; Pc, *Papio cynocephalus*; Ph, *Papio hamadryas*; Tg, *Theropithecus gelada*.


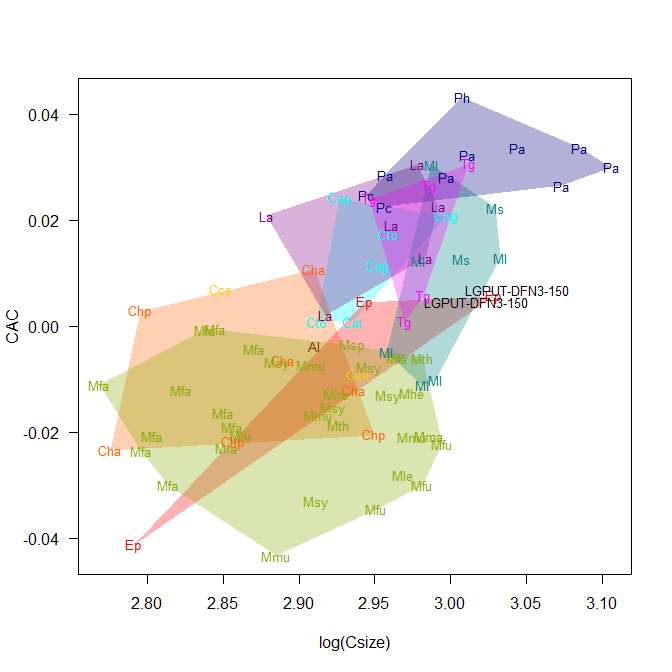

Supplement: Supplementary file 7 — (DOC 65 kb) [file 10764_2022_329_MOESM7_ESM.doc]
