# Supplementary material for: Morphology of the Bony Labyrinth Supports the Affinities of Paradolichopithecus with the Papionina
Source: Int J Primatol. 2022 Sep 20;44(1):209–36. doi: 10.1007/s10764-022-00329-4 (PMC9931825; doi:10.1007/s10764-022-00329-4)
Supplement: Supplementary file 8 — (DOCX 4308 kb) [file 10764_2022_329_MOESM8_ESM.docx]

**Appendix S7.** Principal component analysis conducted on the Procrustes coordinates of the bony labyrinth of extant Cercopithecinae (*n* = 80), together with the left and right labyrinth of the fossil LGPUT DFN3-150.

**Figure 1.** Scree plot. As usuallly in geometric morphometric analyses, each principal component represents only a small proportion of the total variance.


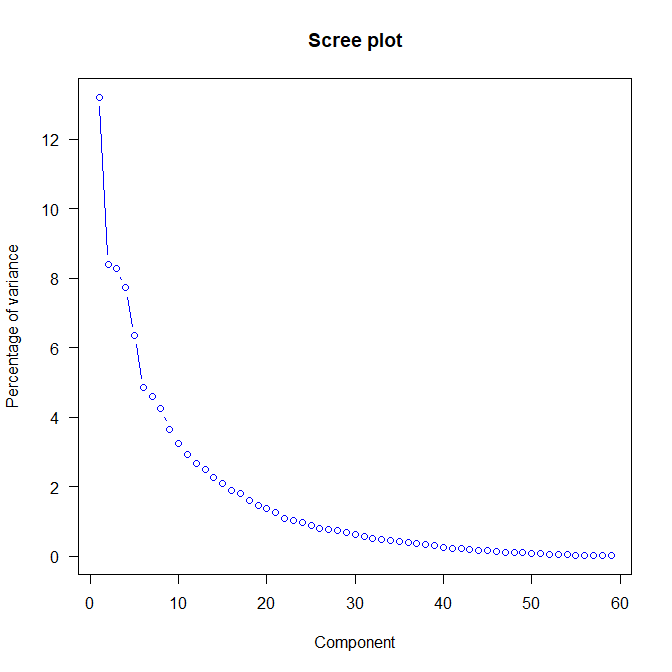


**Figure 2.** First principal components of the Procrustes shape coordinates. (a) PC1 vs. PC2 vs. PC3; (b) PC1 vs. PC2 vs. PC4. The fossil specimen LGPUT DFN3-150 is represented by two large black spheres for the left (L) and right (R) sides. 3D dimensional convex hulls delimit genera. An interactive version of this figure is provided as Appendix S8.


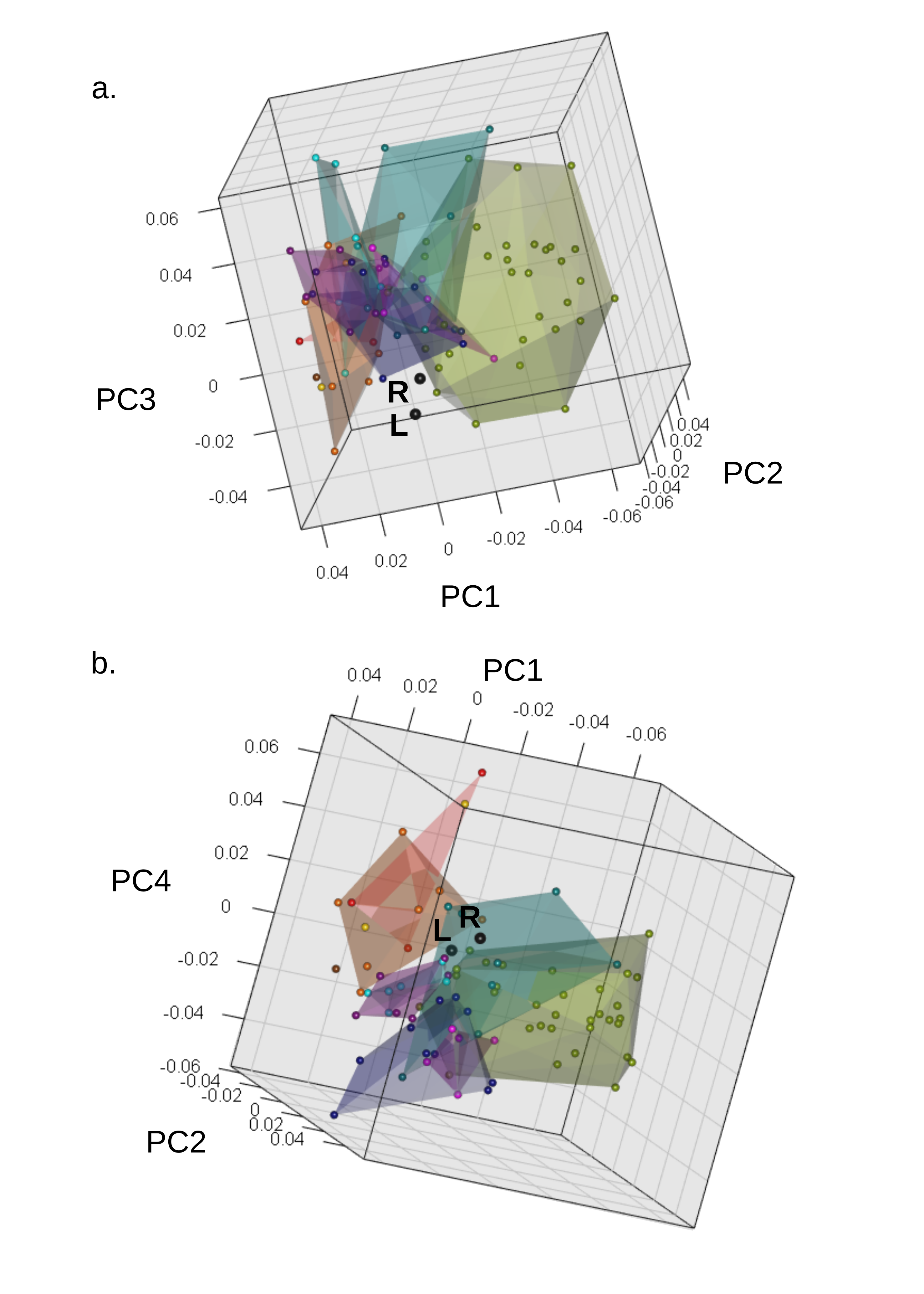


**Figure 3.** Four first principal components (37.6 % of the total variance explained) of the Procrustes shape coordinates of the bony labyrinth in Cercopithecinae. This figure is another version of Fig. 4 of the main text in which species names are displayed.*(a)* PC2 *vs.* PC1; *(b)* PC3 *vs.* PC1; *(c)* PC4 *vs.* PC1.

Al, *Allochrocebus lhoesti*; Cag, *Cercocebus agilis*; Cat, *Cercocebus atys*; Cce, *Cercopithecus cephus*; Cha, *Chlorocebus aethiops*; Chp, *Chlorocebus pygerythrus*; Cto, *Cercocebus torquatus*; Ep, *Erythrocebus patas*; La, *Lophocebus albigena*; Mfa, *Macaca fascicularis*; Mfu, *Macaca fuscata*; Mhe, *Macaca hecki*; Ml, *Mandrillus leucophaeus*; Mle, *Macaca leonina*; Mma, *Macaca maura*; Mmu, *Macaca mulatta*; Mni, *Macaca nigra*; Mra, *Macaca radiata*; Ms, *Mandrillus sphinx*; Msp, *Macaca* sp.; Msy, *Macaca sylvanus*; Mth, *Macaca thibetana*; Pa, *Papio anubis*; Pc, *Papio cynocephalus*; Ph, *Papio hamadryas*; Tg, *Theropithecus gelada*.

(a) PC2 *vs.* PC1


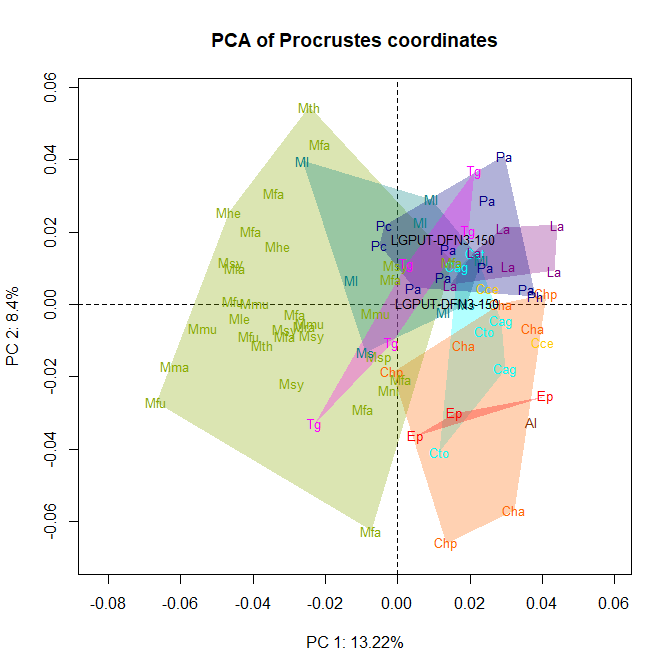


(b) PC3 *vs.* PC1


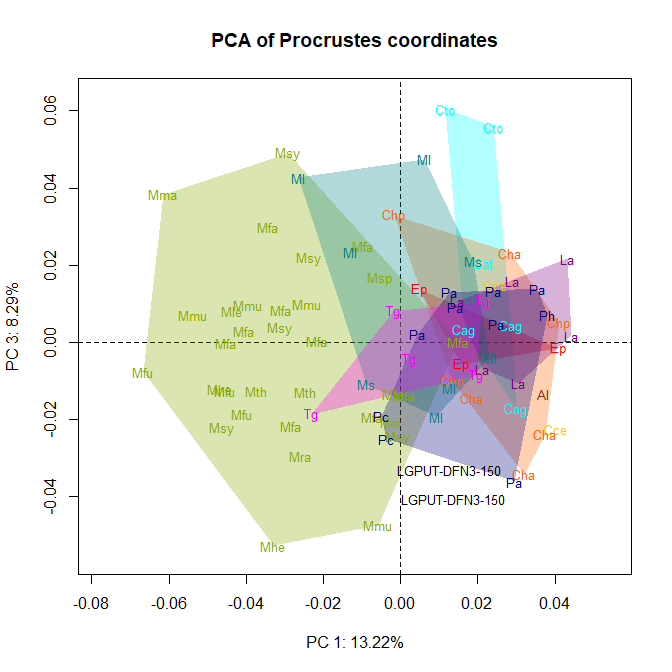
(c) PC4 *vs.* PC1


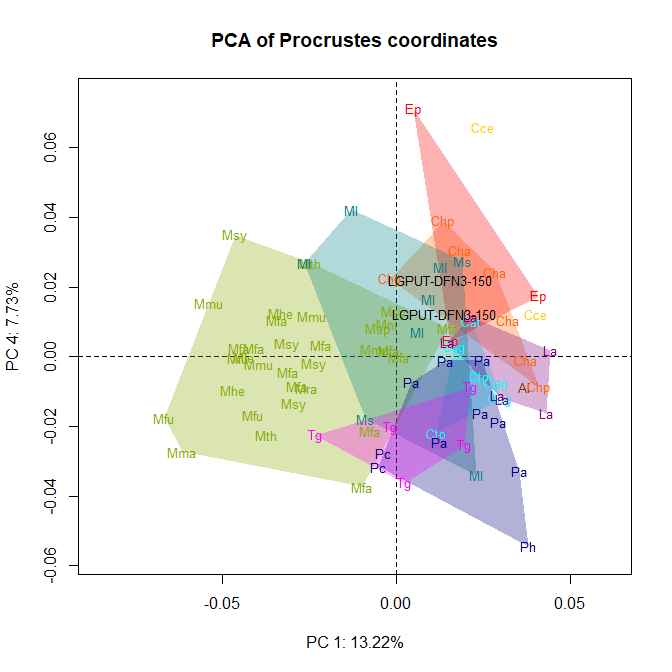


**Figure 4.** Shape variation associated with the four first principal components. For each component, the estimated shape configurations corresponding to the average configuration ±3 standard deviations of the PC scores are represented as warped surfaces (orthographic projections – left, lateral view; right, superior view).


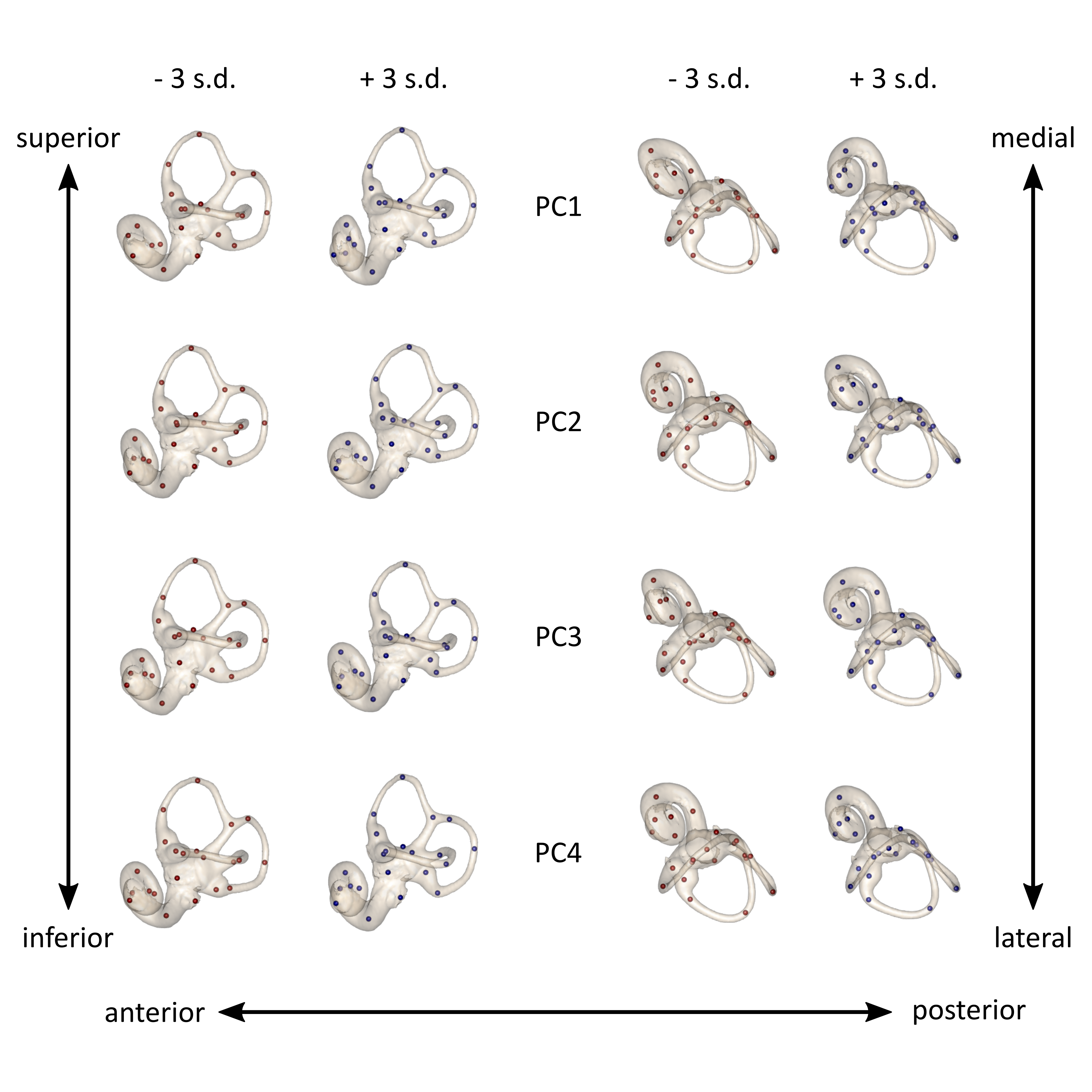
Specimens with high PC1 scores have, relative to the other specimens, an increased torsion of the anterior and lateral semicircular canals (ASC and LSC), whereas their posterior semicircular canal (PSC) is flatter. Because of this, their vertical semicircular canals (ASC and PSC) are rotated posteriorly relative to the axis of maximum elongation of the LSC. The LSC is slightly more posterolaterally and less anterolaterally projected, leading to a less round shape. The vertical canals are more superiorly projected, but with a shorter common crus. Also, the first turn of their cochlea is much less anteriomedially projected, with an increased torsion, and the apex of the cochlear axis points inferolaterally.

Specimens with higher PC2 scores have, relative to other specimens, smaller LSC and PSC, and a flatter PSC. ASC and LSC are more strongly twisted and in opposite directions, so that the position of the vertical canals relative to the plan of the LSC is more superior. The ASC is much higher because its inferior part on the vestibule is more inferiorly positioned. The first turn of the cochlea is flatter, more antero-posteriorly extended and less superiorly projected, and the axis of the cochlea is more horizontal.

Specimens with higher PC3 scores have, relative to other specimens, flatter semicicular canals, with smaller and less inferiorly projected vertical canals (and hence a less round ASC), and a larger and rounder LSC. The cochlea has a flatter first turn, and it is rotated and elongated so that its superior part is more anteromedial and its inferior part more posterolateral.

Specimens with higher PC4 scores have, relative to other specimens, a more inferiorly projected PSC (relative to the LSC), a smaller, less round and more twisted ASC, as well as a rounder, more anteriorly projected and twisted LSC, more inferiorly positioned compared to the PSC. The base of the cochlea is less twisted, and its axis is less horizontal.
