# Supplementary material for: Morphology of the Bony Labyrinth Supports the Affinities of Paradolichopithecus with the Papionina
Source: Int J Primatol. 2022 Sep 20;44(1):209–36. doi: 10.1007/s10764-022-00329-4 (PMC9931825; doi:10.1007/s10764-022-00329-4)
Supplement: Supplementary file 9 — (HTML 1144 kb) [file 10764_2022_329_MOESM9_ESM.html]

Appendix S8


# Appendix S8

Interactive 3D visualisation of the scores for the principal components of the Procrustes shape coordinates of all Cercopithecinae. The fossil specimen *Paradolichopithecus* aff. *arvernensis* LGPUT DFN3-150 is represented by the large black spheres. Three-dimensional convex hulls delimit genera. See legend of Fig. 3 in the main text for the color code.

**Figure 1.** Scores for PC1 vs. PC2 vs. PC3

**Figure 2.** Scores for PC1 vs. PC2 vs. PC4
