# Supplementary material for: Morphology of the Bony Labyrinth Supports the Affinities of Paradolichopithecus with the Papionina
Source: Int J Primatol. 2022 Sep 20;44(1):209–36. doi: 10.1007/s10764-022-00329-4 (PMC9931825; doi:10.1007/s10764-022-00329-4)

**Appendix S9.** Results of the analyses conducted on the residuals of the regression of Procrustes shape coordinates on the log centroid size of the bony labyrinth of extant cercopithecines. Figure 1 and figure 2 correspond to the PCA conducted on the regression residuals, and figure 3 corresponds to a bgPCA conducted on the six first PCs of the regression residuals. The symbols and color code are the same as in the figures in the main text.

**Figure 1.** Scree plot of the principal component analysis of the regression residuals. Each PC describes only a very little proportion of the total variance: the first four and ten PCs explain respectively 37.5 % and 64.6 % of the total variance.


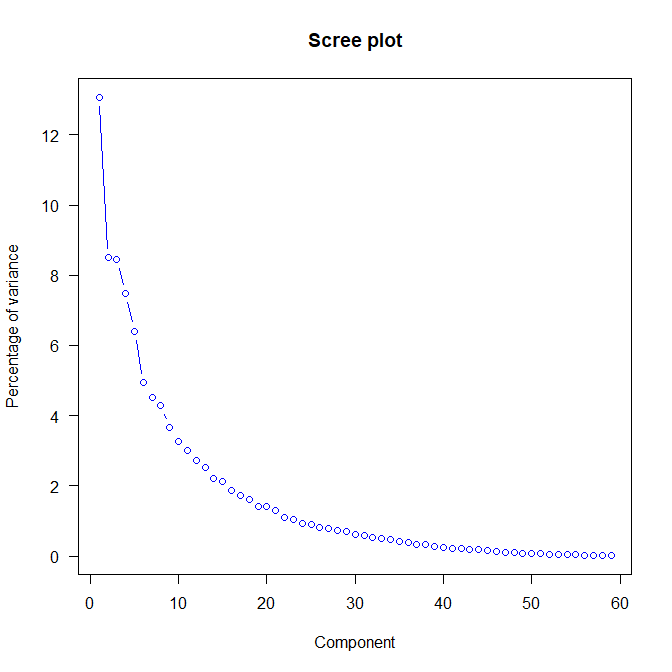


**Figure 2.** Four first principal components (37.5 % of the total variance explained) of the regression residuals.(a), PC2 *vs.* PC1; (b), PC3 *vs.* PC1; (c), PC4 *vs.* PC1. The distribution of the specimens is very similar to their distribution for the corresponding principal components of Procrustes shape coordinates, except for the opposite signs of all PC scores, and that more Macacina tend to overlap with Papionina along PC1 when regression scores are used.


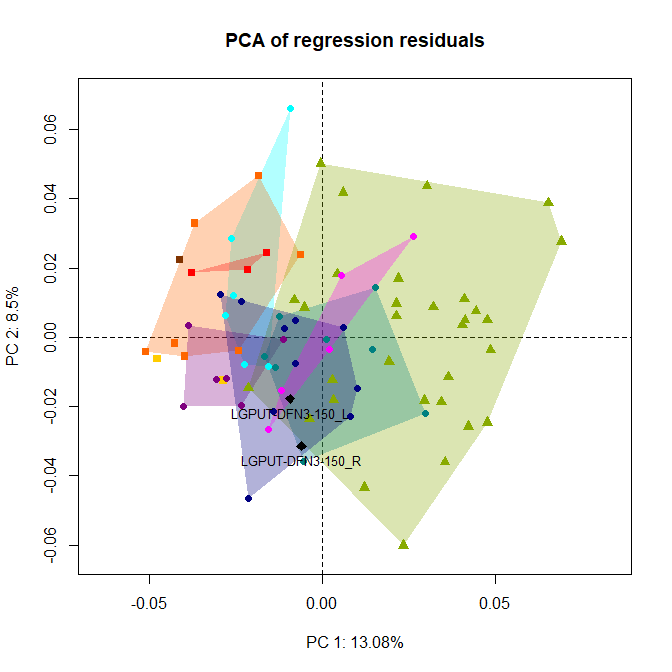


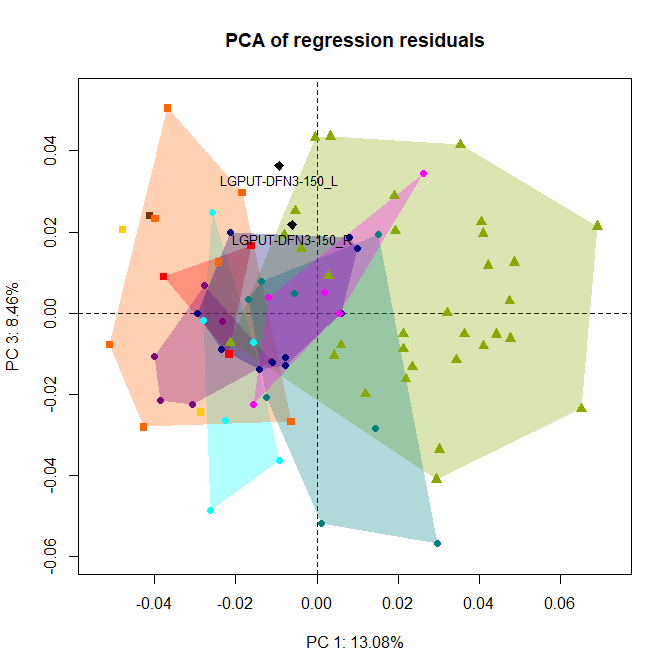


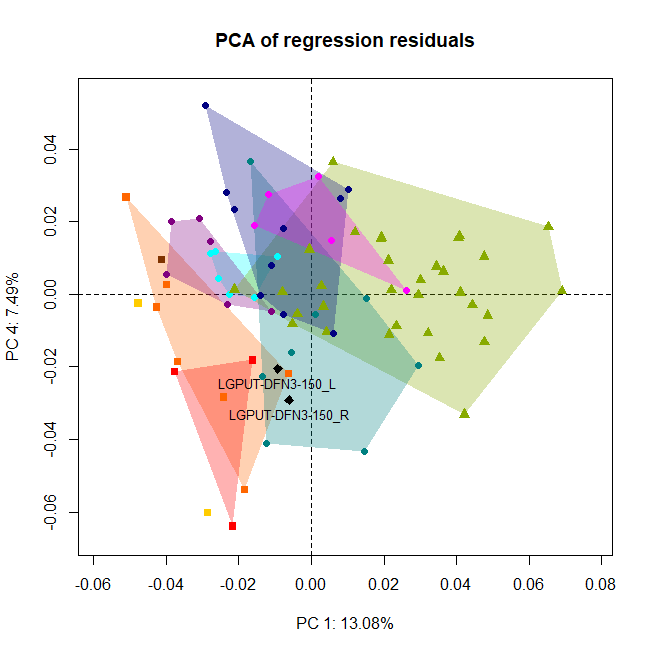


**Figure 3.** Between-group principal components (bgPC) of six allometry-free shape variables describing the bony labyrinth of extant cercopithecines. The shape variables were the first six principal components of regression scores. Convex hulls enclose each group used for the classification, and the black diamonds corresponds to the projection of the left (L) and right (R) labyrinths of the fossil LGPUT DFN3-150 in the space of bgPC1 and bgPC2. The left panel represents the scores for bgPC1 (80.1 % of the between-group variance explained) vs. bgPC2 (19.9 % of the between-group variance explained). The right panel represents the cross-validated scores, for which the three groups tend to overlap more.


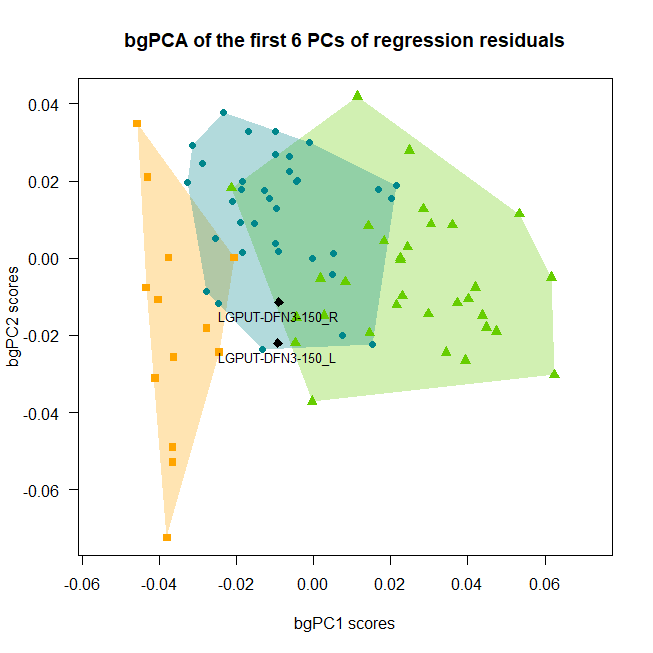

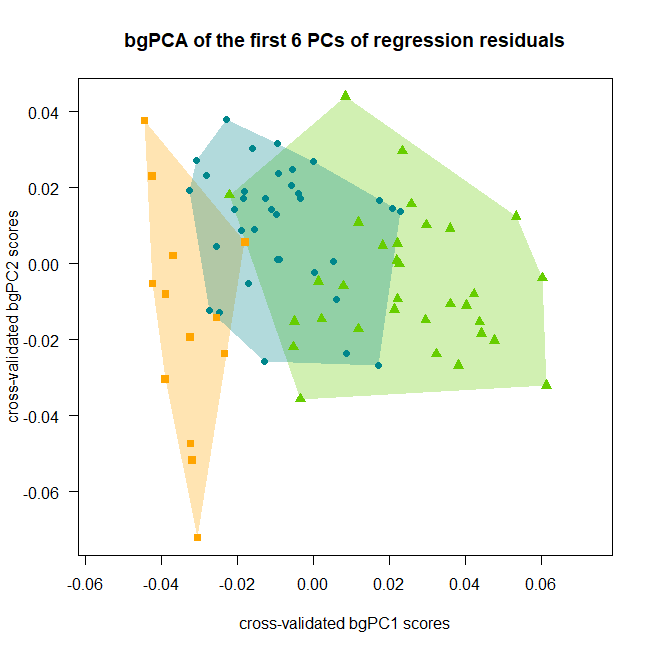

Supplement: Supplementary file 10 — (DOCX 79 kb) [file 10764_2022_329_MOESM10_ESM.docx]
