# Supplementary material for: Morphology of the Bony Labyrinth Supports the Affinities of Paradolichopithecus with the Papionina
Source: Int J Primatol. 2022 Sep 20;44(1):209–36. doi: 10.1007/s10764-022-00329-4 (PMC9931825; doi:10.1007/s10764-022-00329-4)

**Appendix S10.** Between-group principal components (bgPC) of seven shape variables describing the bony labyrinth of extant cercopithecines. The shape variables were the first seven principal components of Procrustes shape coordinates. *(a)* bgPC scores ; *(b)*, cross-validated bgPC scores. This figure is another version of Fig. 5 of the main text in which species names are displayed.

Al, *Allochrocebus lhoesti*; Cag, *Cercocebus agilis*; Cat, *Cercocebus atys*; Cce, *Cercopithecus cephus*; Cha, *Chlorocebus aethiops*; Chp, *Chlorocebus pygerythrus*; Cto, *Cercocebus torquatus*; Ep, *Erythrocebus patas*; La, *Lophocebus albigena*; Mfa, *Macaca fascicularis*; Mfu, *Macaca fuscata*; Mhe, *Macaca hecki*; Ml, *Mandrillus leucophaeus*; Mle, *Macaca leonina*; Mma, *Macaca maura*; Mmu, *Macaca mulatta*; Mni, *Macaca nigra*; Mra, *Macaca radiata*; Ms, *Mandrillus sphinx*; Msp, *Macaca* sp.; Msy, *Macaca sylvanus*; Mth, *Macaca thibetana*; Pa, *Papio anubis*; Pc, *Papio cynocephalus*; Ph, *Papio hamadryas*; Tg, *Theropithecus gelada*.

(a) bgPC scores


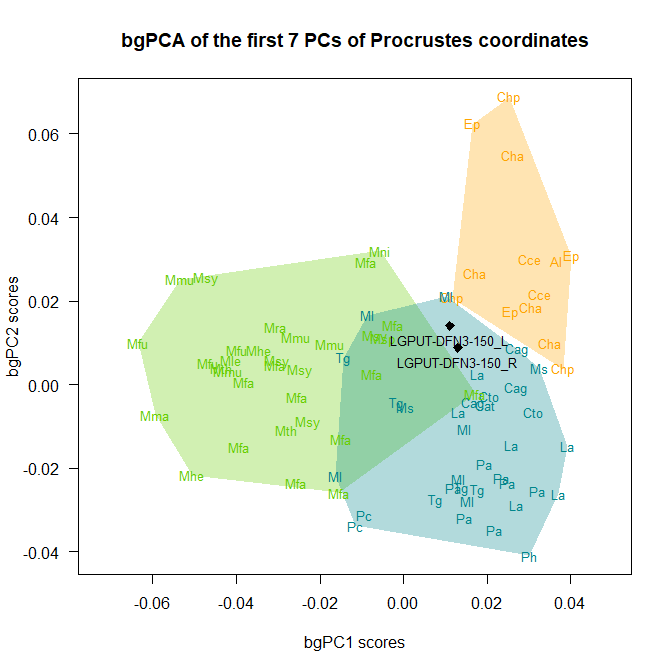


(b) Cross-validated bgPC scores


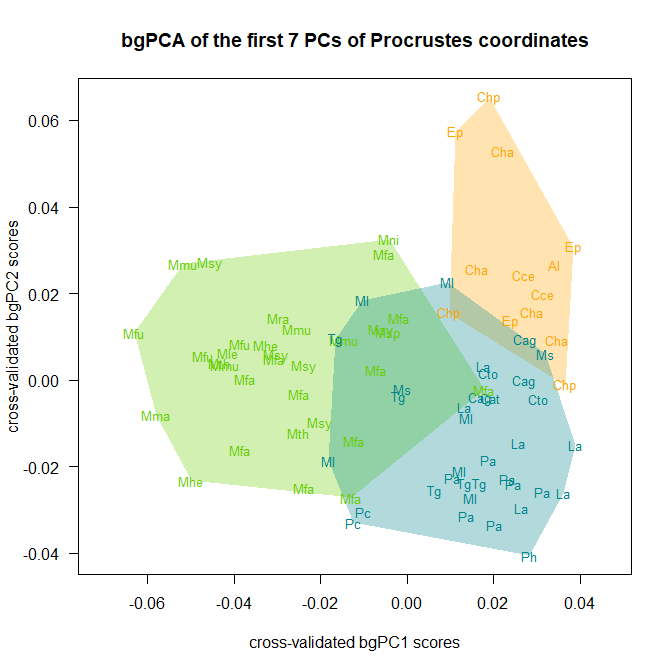

Supplement: Supplementary file 11 — (DOC 78 kb) [file 10764_2022_329_MOESM11_ESM.doc]
